# An approach based on the total‐species accumulation curve and higher taxon richness to estimate realistic upper limits in regional species richness

**DOI:** 10.1002/ece3.3570

**Published:** 2017-11-29

**Authors:** Stanislao Bevilacqua, Karl Inne Ugland, Adriana Plicanti, Danilo Scuderi, Antonio Terlizzi

**Affiliations:** ^1^ Laboratory of Zoology and Marine Biology Department of Biological and Environmental Sciences and Technologies University of Salento Lecce Italy; ^2^ Department of Marine Biology University of Oslo Oslo Norway; ^3^ I.I.S.S. “Ettore Majorana” Catania Italy; ^4^ Department of Life Sciences University of Trieste Trieste Italy; ^5^ Stazione Zoologica Anton Dohrn Napoli Italy; ^6^ CoNiSMa Piazzale Flaminio, 9, Roma Italy

**Keywords:** β‐diversity, habitat mapping, higher taxa, Mollusca, multivariate dispersion, species‐accumulation curves

## Abstract

Most of accumulation curves tend to underestimate species richness, as they do not consider spatial heterogeneity in species distribution, or are structured to provide lower bound estimates and limited extrapolations. The total‐species (T–S) curve allows extrapolations over large areas while taking into account spatial heterogeneity, making this estimator more prone to attempt upper bound estimates of regional species richness. However, the T–S curve may overestimate species richness due to (1) the mismatch among the spatial units used in the accumulation model and the actual units of variation in β‐diversity across the region, (2) small‐scale patchiness, and/or (3) patterns of rarity of species. We propose a new framework allowing the T–S curve to limit overestimation and give an application to a large dataset of marine mollusks spanning over 11 km^2^ of subtidal bottom (W Mediterranean). As accumulation patterns are closely related across the taxonomic hierarchy up to family level, improvements of the T–S curve leading to more realistic estimates of family richness, that is, not exceeding the maximum number of known families potentially present in the area, can be considered as conducive to more realistic estimates of species richness. Results on real data showed that improvements of the T–S curve to accounts for true variations in β‐diversity within the sampled areas, small‐scale patchiness, and rarity of families led to the most plausible richness when all aspects were considered in the model. Data on simulated communities indicated that in the presence of high heterogeneity, and when the proportion of rare species was not excessive (>2/3), the procedure led to almost unbiased estimates. Our findings highlighted the central role of variations in β‐diversity within the region when attempting to estimate species richness, providing a general framework exploiting the properties of the T–S curve and known family richness to estimate plausible upper bounds in γ‐diversity.

## 1 INTRODUCTION

Traditional methods to estimate species richness do not take into account spatial heterogeneity in species distribution within the area of interest, yet it is crucial to model species accumulation as the ensuing estimates could be, in turn, strongly influenced (Chazdon, Colwell, Denslow, & Guariguata, 1998; Colwell & Coddington, 1994; Colwell, Mao, & Chang, 2004; Gotelli & Colwell, 2001). In most cases, conventional accumulation curves overcome this issue by assuming substantial homogeneity within the investigated area. However, if this assumption may be reasonably accepted for local‐scale estimations (Colwell & Coddington, 1994), it might be unrealistic when estimating species richness at a regional scale (i.e., γ‐diversity) or in areas characterized by habitat mixtures. In such contexts, environmental changes across the area are expected to modify the distribution and identity of species composing assemblages from one place to another (Matias, Underwood, Hochuli, & Coleman, 2011). Ignoring these nondirectional variations in β‐diversity (sensu Anderson et al., 2011) constrains the application of classic species accumulation curves to very local contexts and may lead to underestimated species richness extrapolated over large areas (O'Dea, Whittaker, & Ugland, 2006; Reichert et al., 2010; Ugland, Gray, & Ellingsen, 2003).

Despite nonparametric estimators of species richness (e.g., Chao and Jackknife estimators; see Gotelli & Chao, 2013 for a review) allow taking into account spatial heterogeneity, they are sensitive to shifts in species‐abundance distribution (Gwinn, Allen, Bonvechio, Hoyer, & Beesley, 2016) and mainly structured to provide lower bound estimates of species richness at local scale (Gotelli & Colwell, 2001; Shen, Chao, & Lin, 2003). Same considerations apply when estimates are obtained by fitting asymptotic models (e.g., negative exponential or Michaelis–Menten functions; reviewed by Tjørve, 2003) to the smoothed sample‐based accumulation curve, because large areas likely accumulate species at a constant or even an increasing rate due to environmental changes supporting distinctive species assemblages (Gotelli & Colwell, 2011). Improvements from mixture models (Colwell et al., 2004) solved only partially the issue, as they are generally effective for extrapolations over a limited spatial extent, which is often not sufficient to cover the area of interest (Chao, Colwell, Lin, & Gotelli, 2009). Nonasymptotic models, such as the semi‐log model or the power law, are more prone to extrapolations over a large number of samples and produce less conservative estimates of species richness (Tjørve, 2003), but largely disregard spatial heterogeneity.

Ugland et al. (2003) proposed a new approach for estimating species richness at a regional scale in which an overall semi‐log model, namely the total–species (T–S) curve, is fitted to the endpoints of a set of species accumulation curves from distinct spatial units within the total area of study. In contrast to traditional methods, this procedure provides an attempt to model species accumulation accounting simultaneously for variations in species composition among individual samples and potential heterogeneities in species identities among spatial units within the total area sampled. Evidence from study areas where the total species richness was actually known highlighted that the T–S curve provided the most accurate estimate of total richness out of a suite of classical estimation methods, which in most cases produced large underestimations (e.g., O'Dea et al., 2006; Reichert et al., 2010). Yet, doubts still remain about the tendency of the approach to overestimate species richness (Hortal, Borges, & Gaspar, 2006), depending on patterns of commonness and rarity of the species involved (Reichert et al., 2010) and/or the extent to which selected spatial units used in the accumulation model capture true patterns of variation in β‐diversity within the total area (O'Dea et al., 2006).

Understanding whether accumulation curves give realistic estimates of species richness is difficult, if not impossible, in the absence of reliable boundaries. Alternative thresholds, to serve as reference, can be nevertheless derived from higher taxon richness. The actual total number of families in a given region, for instance, can be readily available from baseline checklists. As both β‐diversity and coefficients of T–S curves are strongly correlated across the taxonomic hierarchy up to family level (Terlizzi et al. 2009, 2014), it is expected that variations in β‐diversity within a given area will affect estimates of species and family richness from T–S curves in the same way. In this framework, the performance of the T–S curve may be assessed using families, and improvements leading to realistic estimates of family richness, that is, not above the maximum possible richness, can be considered as conducive also to improved estimates of species richness.

Here, we employed simulated communities and real data on marine mollusk assemblages from three different habitats to show how spatial heterogeneity and rarity of species may affect estimates from T–S curves and, using known total family richness as reference, to demonstrate that the progressive inclusion of such factors in the underlying accumulation model may lead to realistic estimates of family richness. The aim is to reveal some properties of the T–S curve in order to provide a framework to extrapolate species richness over large areas while controlling for potential overestimation not exceeding plausible limits and, therefore, producing estimates that could be considered as potential upper bounds.

## 2 MATERIALS AND METHODS

### 2.1 Study area and dataset

The study area is located along the south Adriatic coast of Apulia (SE Italy, Mediterranean Sea) with a coastline spanning approximately over 20 km. Seven subareas, selected as distinct spatial units based on geomorphological features, habitat distribution, and human activities (Fig. S1), were sampled during a 4‐year monitoring program carried out from 2010 until 2013. Two subareas (S1, S2) had a surface of 1 km^2^, whereas the four remaining subareas (S3–S7) extended over 2 km^2^. Each subarea from S1 to S4 accounted for two habitats, namely rocky reefs and *Posidonia oceanica* seagrass beds, whereas subareas 5–7 were characterized only by coralligenous outcrops (see Fig.S1, see also Table S1 in supplementary material for further details). Benthic assemblages from each habitat within each subarea were sampled at a total of eight randomly selected sampling stations (4 m^2^ surfaces of sea bottom) for larger subareas (two sampled stations per year, from 2010 to 2013), and at four stations (one sampled station per year) for smaller ones (Table S1). In each time of sampling, three samples were collected in each station, for a total of 216 samples. Benthic assemblages were sampled within 0.25 m^2^ squares collecting sediments, and/or scraped rock, within 1‐mm mesh bags using an airlift. Macrofauna was then hand‐sorted under magnification and identified at the finest taxonomic resolution as possible. We focused on mollusks, the most widespread and diverse phylum present, for which all individuals were identified down to the species level. A total of 220 species, belonging to 85 families, were recorded (Table S2 and Appendix S6).

We checked that spatial (i.e., among subareas and habitats) variations in β‐diversity were not confounded by temporal changes in β‐diversity, in order to legitimate the use of samples from different years as a whole set of data to build species‐accumulation curves. Tests on multivariate dispersion (PERMDISP, Anderson, 2006) were carried out separately for each habitat in each subarea, to exclude substantial effects of time in modifying spatial patterns of β‐diversity in the whole sampled area (see Table S3).

### 2.2 Maximum number of families in the area

**Figure 1:**
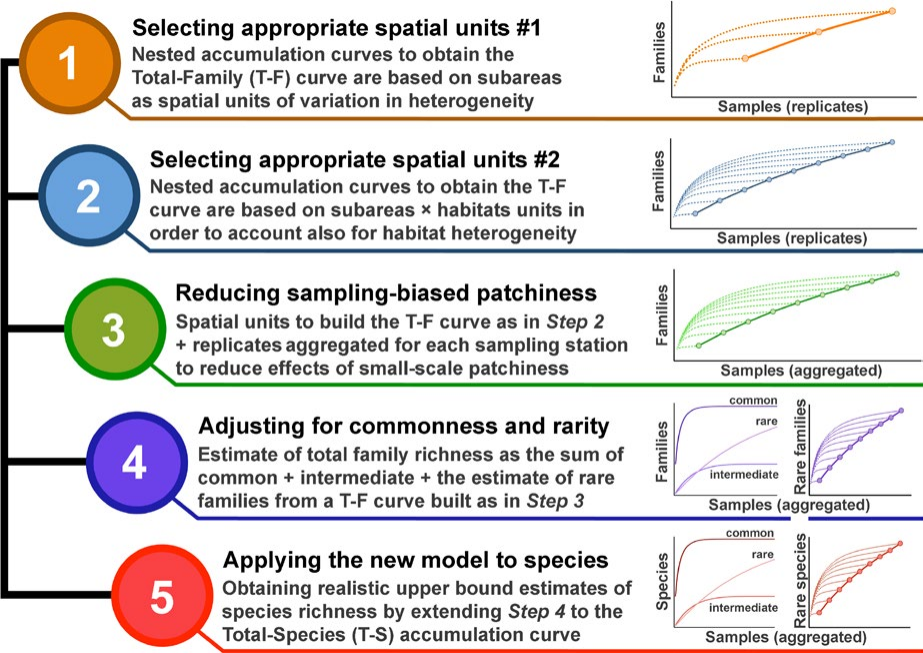
Logical sequence of the stepwise procedure described in the Methods section, which progressively integrate the T–F curve to account for spatial heterogeneity among subareas, among habitats within subareas, small‐scale patchiness, and rarity (Steps 1–4). The last step (5) refers to the application of the fully improved model to species richness estimates

The inventory of marine mollusk families was mined from the literature combining several checklists at regional and basin scale. Families of marine mollusks virtually absent from the investigated marine benthic habitats (because peculiar of deep‐sea habitats, brackish waters, or planktonic and pelagic compartments) were then excluded leading to a total of 242 mollusk families potentially present in the area (Table S4). This number was considered as the highest possible number of families, and used as upper limit to compare against family richness estimated from (1) the T–S curve simply accounting for spatial heterogeneity among subareas, and T–S curves that overcome progressively also potential overestimation due to (2) habitat heterogeneity within subareas, (3) small‐scale patchiness, and (4) patterns of commonness and rarity of taxa. Improvements leading to estimates of total family richness not exceeding the highest possible value were considered as conducive to more reliable estimates of total species richness (Figure 1).

### 2.3 Quantifying β‐diversity within the study area

As a preliminary step, PERMDISP was employed to check whether heterogeneity in composition of mollusk assemblages at species and family level actually varied among subareas and habitats within the total area. Tests were based on Jaccard's distance matrices among samples, with 999 permutations. The design for the analyses consisted of two crossed factors, namely subareas (seven levels, fixed) and habitat (three levels, fixed). We anticipated that patterns of β‐diversity significantly differed among subareas and habitats, consistently between species and families (see Results).

### 2.4 Estimating family richness based on T–S accumulation curve

The T–S accumulation curve (Ugland et al., 2003) allows accounting for heterogeneity among spatial units within the total area. As a first step to build the T–S curve, one species accumulation curve is obtained by randomizing samples of all combinations of 1, 2, 3,…, *n* spatial units, with a given number of random draws of samples for each combination. Then, the overall T–S curve is obtained by fitting a semi‐log model (i.e., number of species vs. the natural logarithm of the sampled area) to the endpoints of the nested species‐accumulation curves (see Ugland et al., 2003 for further details). Ordinary least square regression gives an estimate for the intercept, ${\hat{\mu }}_{S}$, and also for the slope coefficient, ${\hat{\beta }}_{S}$, in the model, and the estimate of the total number of species in the total area of interest, ${\hat{S}}_{\mathrm{Tot}}$, is given by:

(1) $${\hat{S}}_{\mathrm{Tot}}={\hat{\mu }}_{S}+{\hat{\beta }}_{S}\;\left(\ln N\right)$$

where *N *=* A/a*, that is, the number of samples required to cover the whole area of interest, given the area of the sample *a*, and the total area *A*.

The same procedure, using families instead of species, can be applied to obtain a “total‐family” (T–F) curve, and an estimate of the total number of families, ${\hat{F}}_{\mathrm{Tot}}$, in the total area (see Terlizzi, Anderson, Bevilacqua, & Ugland, 2014) as follows:

(2) $${\hat{F}}_{\mathrm{Tot}}={\hat{\mu }}_{F}+{\hat{\beta }}_{F}\;\left(\ln N\right)$$

As there are no measures of dispersion for estimates from the T‐S curve, bootstrap estimates were obtained in order construct 95%CI. Bootstrapping has been widely applied to assess variability of estimates from the T–S curve (Reichert et al., 2010) and other estimators (e.g., Eren, Chao, Hwang, & Colwell, 2012). In this case, for example, a bootstrap set of samples was obtained by resampling the data with replacement for each combination of 1, 2,…, 6, 7 subareas. This procedure was repeated 100 times to obtain 100 estimates on which the 95%CI was constructed. The same approach was applied to the following steps.

### 2.5 Selecting appropriate spatial units to build accumulation curves

The T–S curve accounts for spatial heterogeneity among spatial units within the total area of interest, which are assumed to be homogeneous. However, partitioning the total area into spatial units not aligned with actual patterns of spatial heterogeneity may influence the slope coefficient (${\hat{\beta }}_{S}$) of the T–S curve (O'Dea et al., 2006) and, as a consequence, the ensuing estimate of total richness. To explore the effect of increasing heterogeneity within the selected spatial units on estimates from the T–S model, 12 datasets of 250 species × 1,200 samples were simulated (Appendix S1 and Appendix S5). Each dataset corresponded to one hypothetical region consisting of four spatial units, with three subunits in each spatial unit. For each region, consider that each subunit had a total surface equal to 100 samples of size 1. A total of 250 species were distributed in each region to simulate different patterns of heterogeneity in species composition and small‐scale heterogeneity within (among subunits) and among spatial units, and different patterns of rarity. Summarizing, we simulated 12 hypothetical regions (datasets) each of them with a total area of 1,200 samples and a total species richness of 250 species, with different patterns of heterogeneity in species distribution and rarity (see Appendix S1 for further details). For each simulated dataset, 10 samples of 100 were randomly selected for each subunit, obtaining a subset of 120 random samples that, in practice, simulated a representative sampling of the hypothetical region. For each simulated dataset, the T–S curve was obtained based on (a) the four spatial units and (b) the 12 spatial units × subunits (i.e., taking into account both heterogeneity among spatial units and subunits), and estimates of species richness from the two approaches were compared. Considering spatial units as homogeneous, when they actually are not, might lead to estimate a higher number of species, with respect to T–S curves built taking into account true heterogeneity within spatial units (see Appendix S1).

Analogously to simulated data, in our real case study where changes in species composition and/or small‐scale heterogeneity (i.e., variations in β‐diversity) among habitats within subareas and among subareas are relevant, each habitat in each subarea, rather than subareas, should be the correct spatial units to account for in the accumulation model. We test this hypothesis on real data by estimating family richness in the total area sampled following the two approaches to spatial unit selection employed to analyze simulated data, and using the maximum number of families as reference. As a first step (Figure 1), we built the T–F curve using subareas (as identified in Fig.S1, see also Table S1 for further details) as spatial units, completely ignoring variation in β‐diversity within subareas due to habitat heterogeneity. Nested accumulation curves were obtained for each combination of 1, 2, …, 6, 7 subareas and using 100 random draws of samples for each combination. Family richness was then estimated in the total area sampled following Equation 2. In this case, the total area *A* is the sum of the bottom surface covered by each considered habitat in each subarea, which amounted approximately to 11,000,000 m^2^ (Table S1), whereas the area of one sample, *a*, was equal to 0.25 m^2^. Then, the accumulation model was built by considering each habitat in each subarea as a separated spatial unit (Figure 1, step 2), obtaining a total of 11 subarea × habitat units (Table S1). Family richness was estimated following Equation 2, but the T–F curve was built based on accumulation curves obtained using 100 random draws of samples for each combination of 1, 2,…, 10, 11 subarea × habitat units (*A* and *a* as above).

### 2.6 Reducing sampling‐biased small‐scale patchiness

Heterogeneity in species composition among samples could be strongly affected by sample grain, especially when individuals or species are spatially aggregated or segregated (due for instance to small‐scale environmental variations or biological interactions), and in relation to the extent to which samples are representative of local species assemblages. This, in turn, may influence the estimates of species richness from accumulation curves because of its effect on patchiness (Chazdon et al., 1998). We used a procedure based on random aggregations to identify the number of original smaller scale samples that should be pooled together in order to quantify adequately species composition of local assemblages (see Anderson & Santana‐Garcon, 2015 and Appendix S2 for further details). A reasonable measure of local species diversity is achieved when pooling at least *n *=* *3 original replicate samples (Appendix S2). Therefore, the three replicates in each station were summed obtaining a total of 72 aggregated samples, and used to build the T–F curve, in order to check whether sample pooling would have reduced overestimation of the total family richness by overcoming potential effects of small‐scale aggregation of species (Gotelli & Colwell, 2011). The T–F curve was built as above, with accumulation curves obtained using 100 random draws of the 72 aggregated samples for each combination of 1, 2,…, 10, 11 subarea × habitat units (Figure 1, step 3). Note here that the cumulative list of families from the three samples was assumed as representative of the family pool in each station, and the area of one sample *a* was considered equal to 4 m^2^ (i.e., the surface of one sampling station).

### 2.7 Adjusting the model for rare, intermediate, and common families

Once habitat heterogeneity within subareas was incorporated in the accumulation model, and the potential effect of small‐scale patchiness fixed, the next step to further improve estimates of family richness focused on adjusting estimates according to rarity of species (Figure 1, step 4). The 85 mollusk families found in the sampled area were classified as common, intermediate, and rare if observed, respectively, in <5%, >5% and <10%, and >10% of the aggregated samples (Gauch, 1982; Reichert et al., 2010; Ugland & Gray, 1982).

As the model in Equation 1, and analogously in Equation 2, is approximately additive, the estimated total number of families ${\hat{F}}_{\mathrm{Tot}}$ in the total area of interest can be considered as the sum of the estimated total number of common ${\hat{F}}_{\mathrm{Tot}}^{\mathrm{Com}}$, intermediate ${\hat{F}}_{\mathrm{Tot}}^{\mathrm{Interm}}$, and rare ${\hat{F}}_{\mathrm{Tot}}^{\mathrm{Rare}}$ families in the area obtained following Equation 2:

(3) $${\hat{F}}_{\mathrm{Tot}}\approx {\hat{F}}_{\mathrm{Tot}}^{\mathrm{Com}}+{\hat{F}}_{\mathrm{Tot}}^{\mathrm{Interm}}+{\hat{F}}_{\mathrm{Tot}}^{\mathrm{Rare}}$$

It is worth noting here that the linear extrapolation of the number of families over the whole area of interest based on the T–F curve implies that richness increases continuously at increasing number of samples. However, if this might be true for rare families, the same could not occur for common and intermediate ones, and the linear extrapolation could overestimate total family richness because it would tend to overestimate the number of common and intermediate families. It is reasonable to assume that most, if not all, of the intermediate and common families in the total area of interest would be detected after a relatively minor proportion of the area has been sampled (see Appendix S3) and, therefore, that their respective accumulation curves would achieve saturation in routine biodiversity surveys, as the present study (see Results). In this view, three accumulation curves can be obtained by considering common, intermediate and rare families separately, and estimates of common (${\hat{F}}_{\mathrm{Tot}}^{\mathrm{Com}}$) and intermediate (${\hat{F}}_{\mathrm{Tot}}^{\mathrm{Interm}}$) families in Equation (3) can be substituted with their observed number in the area of interest, $F_{\mathrm{Obs}}^{\mathrm{Com}}$ and $F_{\mathrm{Obs}}^{\mathrm{Interm}}$, respectively, obtaining:

(4) $${\hat{F}}_{\mathrm{Tot}}\approx F_{\mathrm{Obs}}^{\mathrm{Com}}+F_{\mathrm{Obs}}^{\mathrm{Interm}}+{\hat{F}}_{\mathrm{Tot}}^{\mathrm{Rare}}$$

Analysis of simulated data demonstrated that the additive model in Equation 4 led to improve estimates from the T–S curve under different scenarios of spatial heterogeneity (see Appendix S4). The additive model (Equation 4) was then applied to real data to obtain estimates of family richness. In this case, the T–F curve for rare families was built following the Equation 2, with accumulation curves obtained using 100 random draws of the 72 aggregated samples for each combination of 1, 2,…, 10, 11 subarea × habitat units.

### 2.8 Applying the new model for estimating species richness

The whole stepwise procedure described previously was naturally extended to species‐level data in order to obtain species richness estimates in the study area (Figure 1, step 5).

Three accumulation curves were built considering common, intermediate, and rare species separately. Then, analogously to Equation 4, the total number of species in the total area, ${\hat{S}}_{\mathrm{Tot}}$, is obtained as follows:

(5) $${\hat{S}}_{\mathrm{Tot}}\approx S_{\mathrm{Obs}}^{\mathrm{Com}}+S_{\mathrm{Obs}}^{\mathrm{Interm}}+{\hat{S}}_{\mathrm{Tot}}^{\mathrm{Rare}}$$

where $S_{\mathrm{Obs}}^{\mathrm{Com}}$ and $S_{\mathrm{Obs}}^{\mathrm{Interm}}$ are, respectively, the observed number of common and intermediate species in the area of interest, whereas ${\hat{S}}_{\mathrm{Tot}}^{\mathrm{Rare}}$ is the estimates of rare species from the T–S curve built following the Equation 1, with accumulation curves obtained using 100 random draws of samples (72 stations) for each combination of 1, 2,…, 10, 11 subarea × habitat units.

All analyses reported here and in the previous paragraphs were carried out using R (R Development Core Team, 2016).

## 3 RESULTS

β‐diversity of mollusk assemblages significantly differed among subareas and habitats (Table 1), indicating that neither the whole sampled area nor the subareas are homogeneous, but rather that each habitat in each subarea represented a separate spatial unit in terms of heterogeneity in species composition. Such patterns of variation in β‐diversity were consistent at family level (Table 1).

**Table 1:** Summary of tests for multivariate dispersion (PERMDISP) carried out to check for difference in β‐diversity among groups of replicate samples from different habitats and subareas. dfn = degrees of freedom of the numerator; dfd = degrees of freedom of the denominator

Source of variation	dfn	dfd	Species		Families	
			*F*	*p* (perm)	*F*	*p* (perm)
Subarea	6	209	5.588	.001	2.810	.030
Habitat	2	213	13.407	.001	25.414	.001
Subarea × Habitat	10	205	2.849	.017	4.348	.001

**Figure 2:**
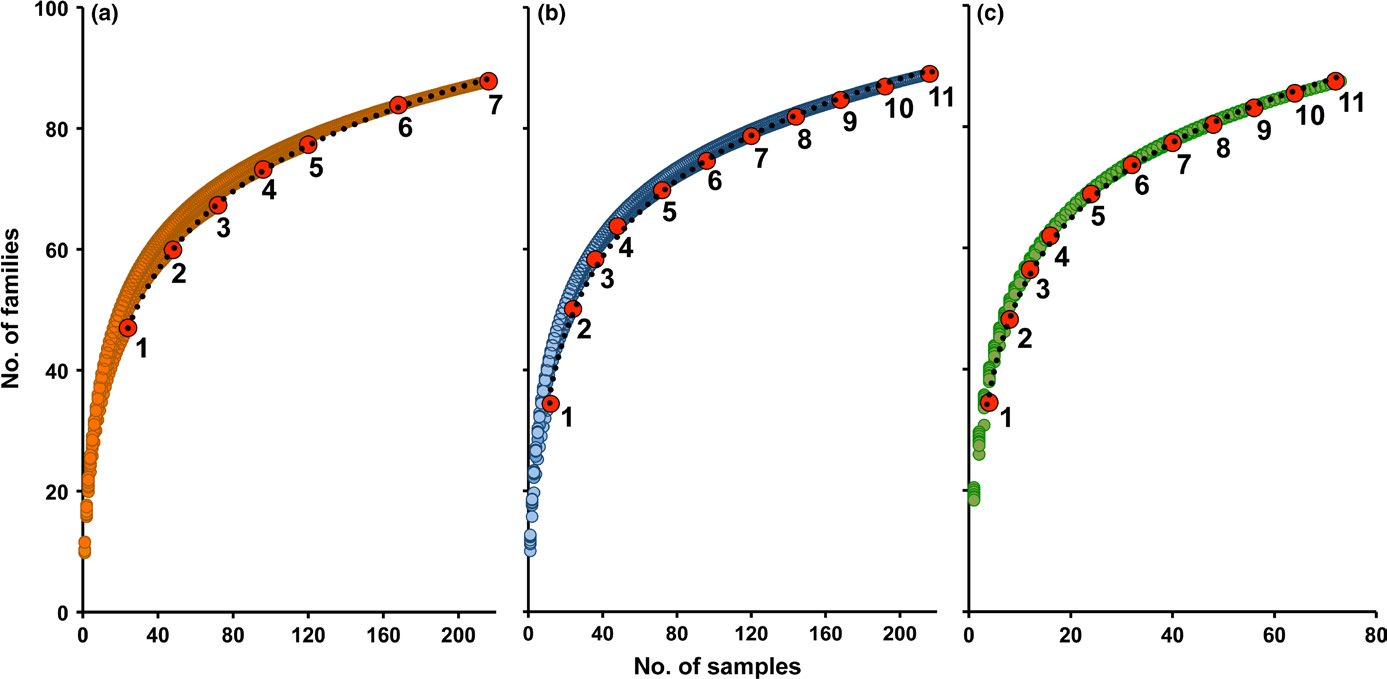
T–F curves accounting progressively for spatial heterogeneity due to (a) subareas only; subareas and habitats (b); subareas, habitats and patchiness (c). The T–F curves (dotted black lines) through the terminal points (red circles) of nested accumulation curves are showed. Nested accumulation curves were obtained for each combination of 1, 2,…, 6, 7 subareas (a) and of 1, 2,…, 10, 11 subareas × habitat units (b, c) within the total area. Replicate samples (0.25 m^2^) were used to build accumulation curves in (a) and (b) (*n *=* *216), whereas in (c), samples (*n *=* *72) were the sum of three replicates in each station (4 m^2^)

**Figure 3:**
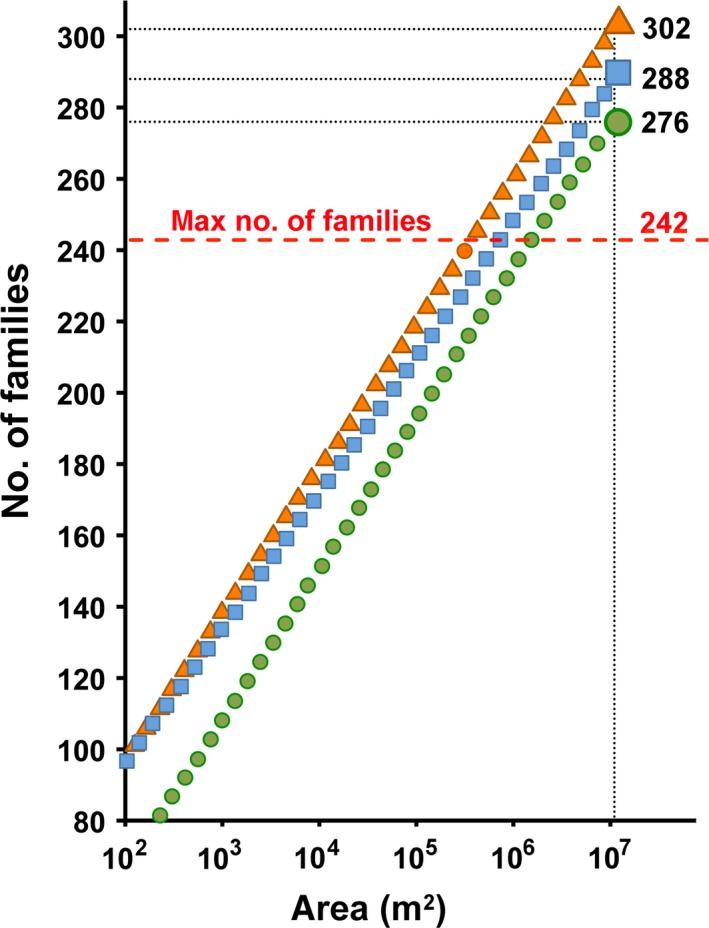
Extrapolation of total family richness (${\hat{F}}_{\mathrm{Tot}}$) over the total area (11 × 10^6^ m^2^) from the T–F curves (dotted lines) accounting for spatial heterogeneity due to subareas only (orange triangles), subareas and habitats (blue squares), subareas, habitats and patchiness (green circles). Note that *x*‐axis is log‐scaled

**Figure 4:**
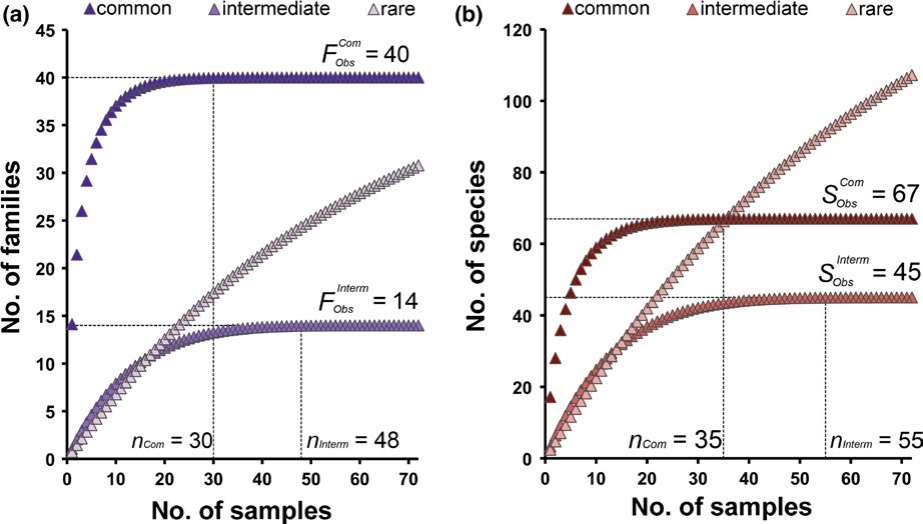
Randomized accumulation curves of common, intermediate, rare families (a) and species (b). The number of common ($F_{\mathrm{Obs}}^{\mathrm{Com}}$) and intermediate ($F_{\mathrm{Obs}}^{\mathrm{Interm}}$) families and species ($S_{\mathrm{Obs}}^{\mathrm{Com}}$, $S_{\mathrm{Obs}}^{\mathrm{Interm}}$) at saturation were reported along with the number of samples to achieve saturation (respectively, *n*_Com_ and *n*_Interm_)

The estimated parameters of the T–F curve (Equation 2) for real data based on subareas (Figure 2a) led to estimate a total number of 302 families (${\hat{F}}_{\mathrm{Tot}}$; Table 2), which largely overestimated (~25%) the maximum possible number of 242 families (Figure 3). The estimate from the T–F curve based on the 11 subarea × habitat units (Figure 2b) was lower (${\hat{F}}_{\mathrm{Tot}}$ = 288), but still exceeded (~19%) this threshold (Table 2, Figure 3). Overestimation still persisted, although further reduced (~14%), when the T–F curve was based on aggregated samples (Figure 2c), which led to a total number of 276 families (Table 2, Figure 3). Randomized accumulation curves showed that the number of common and intermediate families in the area achieved saturation after 30 and 48 aggregated samples, respectively (Figure 4a), indicating that sample size (*n *=* *72) was sufficient to detect all common ($F_{\mathrm{Obs}}^{\mathrm{Com}}$ = 40) and intermediate ($F_{\mathrm{Obs}}^{\mathrm{Interm}}$ = 14) families. As expected, the number of rare families increased continuously as the number of considered samples increased (Figure 4a). The estimated parameters of the T–F curve for rare families based on subarea × habitat units and aggregated samples were provided in Table 2. The calculation of ${\hat{F}}_{\mathrm{Tot}}$ following Equation 4 led to estimate a total number of 183 families (Table 2), which was fairly below the maximum number.

**Table 2:** Estimated parameters of semi‐log models for the T–F curve accounting for spatial heterogeneity (1) among subareas only, (2) subareas and habitats, (3) subareas and habitats but using aggregated samples, (4) subareas and habitats using aggregated samples and the additive model for common, intermediate, and rare families (see Figure 1). Estimates of the total number of families in the sampled area were provided along with upper and lower 95% confidence limits from bootstrap (in brackets). NA = not applicable

T–F curve model	(1) Heterogeneity among subareas	(2) Heterogeneity among subareas and habitats	(3) Heterogeneity among subareas and habitats, and small‐scale patchiness	(4) Heterogeneity among subareas and habitats, small‐scale patchiness, and rarity
Spatial units	7 subareas	11 subarea × habitat units	11 subarea × habitat units	11 subarea × habitat units
Number of samples	216	216	72	72
Slope coefficient (${\hat{\beta }}_{F}$)	17.62	16.45	18.03	9.47
Intercept (${\hat{\mu }}_{F}$)	−8.25	−2.48	8.32	−11.38
*R* ^2^	0.995	0.991	0.998	0.981
Estimated number of rare families (${\hat{F}}_{\mathrm{Tot}}^{\mathrm{Rare}}$)	NA	NA	NA	129 (139, 92)
Number of common families ($F_{\mathrm{Obs}}^{\mathrm{Com}}$)	NA	NA	NA	40
Number of intermediate families ($F_{\mathrm{obs}}^{\mathrm{Interm}}$)	NA	NA	NA	14
Estimated total family richness (${\hat{F}}_{\mathrm{Tot}}$)	302 (329, 270)	288 (322, 266)	276 (299, 257)	183 (193, 146)

The estimated slope coefficient of the T–S curve on real data was ${\hat{\beta }}_{S}$ = 58.79 and the intercept ${\hat{\mu }}_{S}$ = −89.91 (*R* ^2^ = 0.991), in the absence of any adjustment. In this case, the estimated total number of species in the total area (Equation 1) was ${\hat{S}}_{\mathrm{Tot}}$ = 945 (95%CI: 968–845). Using subarea × habitat units to build the T–S curve led to reduce this estimate of ~11%, whereas using aggregated samples led to further reduce the estimated species richness of 18%. Randomized accumulation curves saturated after considering 35 and 55 aggregated samples for common and intermediate species, respectively, indicating that all common ($S_{\mathrm{Obs}}^{\mathrm{Com}}$ = 67) and intermediate ($S_{\mathrm{Obs}}^{\mathrm{Interm}}$ = 45), but not rare, species were sampled (Figure 4b). The estimated parameters of the T–S curve for rare species adjusted to account for habitat heterogeneity within subareas and small‐scale patchiness were ${\hat{\beta }}_{S}^{\mathrm{Rare}}$ = 32.97 and ${\hat{\mu }}_{S}^{\mathrm{Rare}}$ = −39.28 (*R* ^2^ = 0.982), and the fully adjusted model (Equation 5) led to estimate a total number of 562 (95%CI: 570–438) species (${\hat{S}}_{\mathrm{Tot}}$).

## 4 DISCUSSION

Three major sources of heterogeneity may drive patterns of species occurrence in samples from natural communities, thus potentially affecting estimates of regional species richness based on accumulation curves. The first, and perhaps more intuitive, source relates to spatial variations in environmental features (e.g., geographic factors, habitat changes), which may lead composition of species assembly to vary across the investigated area (Gotelli & Colwell, 2011). The other two main drivers of spatial heterogeneity are “patchiness,” which encompasses small‐scale aggregation and segregation of individuals or species, and variations in frequency of occurrence among species due their overall commonness or rarity (Colwell et al., 2004). The stepwise adaptation of the T–S curve to account for these aspects produced a progressive alignment of estimated number of families to their maximum possible richness, leading to realistic estimates (i.e., below this maximum limit) when all the three sources of heterogeneity were considered in the accumulation model. Analyses of simulated communities confirmed our findings on real data, highlighting that almost unbiased estimates were achieved when spatial heterogeneity ranged from medium‐high to very high levels and species with low (5%–10%) to very low (<5%) occurrence in samples did not represent an excessive proportion (>2/3) of all species.

A finer partition of spatial units to be used in accumulation curves, taking into account significant levels of heterogeneity among habitats within subareas, reduced of about 5% the overestimation of the maximum number of families. It is worth noting that accumulation curves at higher taxonomic levels naturally lie below the corresponding species‐level curves (Gotelli & Colwell, 2001) showing less steep patterns of accumulation (Terlizzi et al., 2014). Therefore, relatively small refinements to the estimated family richness turn into more remarkable ones when the finer partition is applied to species accumulation, which in our case led to estimate about 11% less species if compared to the classic model (845 vs. 945, respectively). In addition, the outcomes of including habitat heterogeneity in the accumulation model strongly depend on the magnitude of underlying variations in β‐diversity, which in our case were likely low although statistically significant. In fact, when spatial units used to build the T–S curve are homogeneous, splitting them into subunits according to putative environmental or habitat features has no substantial effects on the ensuing estimates (O'Dea et al., 2006), whereas the potential overestimation may largely increase at increasing heterogeneity within such spatial units, up to >80% more species, as our simulated data has confirmed.

Despite the concept of β‐diversity encompasses also nondirectional changes in species composition among samples within a given spatial extent (Anderson et al., 2011; Chao & Chiu, 2016), which are strongly correlated to patterns of species accumulation (Terlizzi et al., 2014), attempts to estimate regional diversity are rarely associated with explicit assessments of β‐diversity patterns. Our findings stressed the need to quantifying variations in β‐diversity within the area of interest in order to guide the choice of the approach to species richness estimation, understanding whether the assumptions underlying accumulation models are respected and, if applying the T–S model, to identify the correct spatial units to obtain the nested accumulation curves.

The mechanism generating overestimation in the T–S curve relies on its ability to account for spatial heterogeneity by stratifying species accumulation among spatial units within the total area of interest. This peculiarity of the T–S model represents the strength and the weakness of the approach depending on the extent to which the selected spatial units identify actual discontinuities in patterns of β‐diversity. When the area is not homogeneous, the nested structure of the T–S model reflects more closely the true rate of species accumulation within the area, unlike traditional curves that completely ignore spatial heterogeneity and generally lead to underestimate extrapolated species richness (Reichert et al., 2010). This occurs because traditional accumulation curves, by combining samples from different spatially heterogeneous portions of the sampled area, will necessarily lie above a curve that combine progressively an equal number of samples from one, two, three,…, *n* portions of the area, as the T–S curve does (Ugland et al., 2003). On the other hand, the model will tend to overestimate species richness if spatial units defined to build the T–S curve are still spatially heterogeneous entities that can be further partitioned in order to match the true discontinuities in β‐diversity. In this case, the T–S curve will lie below the curve based on the true basic spatial units of variations, leading to overestimated species richness.

Analogous mechanisms underlie the effect of patchiness in modifying the slope of accumulation curves and the ensuing estimates of species richness. If species are randomly distributed across samples, the initial rate of accumulation will be higher with respect to patchy distributions, leading extrapolations from accumulation curves to estimate more species in the latter case (Chazdon et al., 1998; Gotelli & Colwell, 2011). When individuals are spatially aggregated, or species distribution at local scale is nonrandom, sample grain could determine an increase in patchiness, especially when samples have a limited surface if compared to the size of the underlying assemblage (Gotelli & Colwell, 2011). In these contexts, and especially if fine sample grains (such as a 1‐m^2^ plots or smaller) are used, a portion of α‐diversity could be erroneously ascribed to the β component of diversity (Crist & Veech, 2006), with a consequent overestimation of total species richness. Hortal et al. (2006) found, indeed, a low sensitivity of species richness estimators to sample grain, although this property mostly concerned nonparametric estimators and, in the end, could be explained by the fact that the particular community under study (epigean arthropods) was sampled equally well irrespective of sample grains. Unfortunately, attempts to quantify the effect of sample grain on extrapolations from accumulation curves at varying habitat and type of assemblage are still largely lacking (Drakare, Lennon, & Hillebrand, 2006), and empirical assessments of this effect are difficult without reliable reference thresholds of total species richness. In this respect, our approach could help discerning undesirable influences of sample grain, guiding the decision to aggregate smaller scale samples into larger ones if conducive to reduce overestimation (Anderson & Santana‐Garcon, 2015).

Amendments to the T–S model to account for habitat heterogeneity and patchiness were not sufficient to prevent the overestimation of family richness beyond the maximum possible number of 242 families. The estimated number of families in the investigated area fell definitely below this threshold only after the inclusion of rarity in the model, which led to estimate a total of 183 families. This is not surprising, as the proportion of common and rare taxa may strongly affect accumulation curves and the ensuing estimated richness, especially for highly diverse groups of organisms (Longino, Coddington, & Colwell, 2002). As many other accumulation curves, with the exception of some nonparametric estimators (e.g., ACE, ICE; Chao & Lee, 1992; Chazdon et al., 1998), the T–S curve does not consider the proportion of rare and common species within the investigated area, and is likely to perform better when the probability of encountering rare species is neither high nor low (Reichert et al., 2010). Corrections to the estimated total richness are difficult to be carried out as the rate of occupancy of rare taxa within a given area is generally unknown, and its estimates largely biased unless an extremely intensive sampling effort is carried out. However, partitioning the contribution of common, intermediate, and rare taxa allows amending the overestimation of the linear extrapolation irrespective of patterns of spatial heterogeneity (see Appendix S4), at least for common and intermediate taxa when sampling efforts are sufficient to allow their saturation, as occurred in our study and likely the case in most of current biodiversity assessments.

Improvements deriving from family‐level curves to species accumulation led to estimate a total of 562 species. The fact that the species recorded by sampling a tiny fraction of the total area (0.000005%) were >39% of the estimated number seems to indicate this estimate as reasonable, also because it referred to a highly speciose phylum of marine invertebrates from three different habitats, two of them, namely coralligenous outcrops and *P. oceanica* meadows, among the most diverse in the Mediterranean (Ballesteros, 2006), within a region at the intersection between two biogeographic zones (i.e., the Adriatic and the Ionian Sea). The number of species estimated using the classic approach (945), instead, looks excessive and would imply that 2/3 of all species of marine mollusks known for the whole Italian coast were putatively present in the study area. Although these considerations could appear rather speculative in the absence of reliable information about the true number of species, evidence from family‐level accumulation curves and simulated data demonstrated that the estimate from the classic T–S curve was largely biased toward overestimation and potentially leading to estimate >65% more species. The application of other estimators to our data produced incongruent estimates of species and family richness that were unreasonably high for nonasymptotic parametric estimators (e.g., power law model) or very close, if not below, to the observed number of taxa for asymptotic ones (e.g., negative exponential model), and only nonparametric estimators (e.g., Chao2) predicted acceptable values (see Table S5). It is worth stressing here once again, however, that nonparametric estimators focus on finding how many species may have been in a set of samples (Colwell & Coddington, 1994), thus providing a conservative estimate that predict how many species might be present at least. Although these estimators account for spatial heterogeneity in species composition, they do not operate to extrapolate the number of species that may have been if the whole area of interest would have been sampled or, at the best, they allow extrapolations over two‐three times the number of original samples (e.g., Colwell et al., 2004). In contrast, fitting a given model to species accumulation allows extrapolations over large areas, but these estimators largely neglect spatial heterogeneity and, depending on the selected model, often lead to severe under‐ or overestimation (Hortal et al., 2006; O'Dea et al., 2006; Reichert et al., 2010; Ugland et al., 2003). Only the T–S curve combines the possibility to extrapolate over large areas with an accumulation model structured to account for heterogeneity among samples and among different spatial units.

A major problem when determining the reliability of species richness estimators relies on the fact that in most cases neither the actual species richness nor the species‐abundance distribution in a given area are known, and the best that can be done is to obtain upper and lower bounds on species richness (O'Hara 2005). However, if the use of nonparametric estimators could be an effective solution for reliable lower bound estimates of species richness (Gotelli & Colwell, 2001), the identification of superior limits is more problematic (O'Hara 2005). As stated by the statistician I. J. Good, and reported in Bunge & Fitzpatrick (1993, p. 370), it is usually not possible to estimate the number of unseen species, as there is nearly always a very large number of rare species and, under a wide range of models, only lower bounds are identifiable (Mao & Lindsay 2007). Attempts to use maximum known limits to set upper bound estimates has been performed in other field of research, such as in estimating the number of archaeological artefacts (Eren et al., 2012), but the approach is not applicable to most biodiversity research due to the lack of suitable references for the maximum limits in species richness. In the general absence of theoretical and empirical bounds, the use of upper limits derived from higher taxon richness could represent a profitable strategy, as their number may be considered almost fixed for many groups of organisms, at least over the genus level (Mora, Tittensor, Adl, Simpson, & Worm, 2011), and merit further investigations to understand its potential application to a wide range of estimators. To date, the T–S curve represents a unique estimator in which the abovementioned desirable properties add to the peculiarity of the accumulation coefficient to intimately relate across the taxonomic hierarchy up to family level (Terlizzi et al., 2009, 2014). Such prerogatives make this estimator eligible to explore refinements referring to known upper limits in family richness, and allow assuming that the ensuing estimates could be more aligned with realistic upper bounds also at species level.

An upper bound should be (1) greater than or equal to the true value, but it should be (2) lower than or equal to the maximum possible value of richness, including its confidence interval. Nonasymptotic parametric estimators, such as the T–S curve, are intrinsically prone to overestimate the true richness although, as occurs for the other estimators, a negative bias is possible for hyper‐diverse communities with many rare species, or when the region of interest is severely under‐sampled. This issue was analyzed in detail by Reichert et al. (2010), showing that the T–S curve will underestimate only when the probability of being kept is vanishingly small for a very large portion of species or, in other words, when most of species in the community under study are extremely rare. Despite no univocal consensus has been achieved around the general model best fitting species‐abundance distributions, it is nevertheless quite clear that this model in real‐world communities is likely to be a symmetrical one (e.g., log‐normal; Alroy, 2015; Ulrich, Ollik, & Ugland, 2010), implying that extremely rare species are equally numerous as less rare/common species. Also, even in presence of truly left‐skewed species‐abundance distributions, the portion of extremely rare species (one or few individuals) is a relatively minor component of the total number of species (e.g., McGill et al., 2007). In the other cases, skewness of the left side of species‐abundance distributions is only apparent, due to Preston's veil line (Preston, 1948) or peculiar conditions, such as high immigration rates, or presence of transient species (McGill et al., 2007). The tendency of the T–S curve to exceed the true values was also empirically showed in several studies where the true richness was actually known (Hortal et al., 2006; O'Dea et al., 2006; Reichert et al., 2010), and confirmed by our simulations. Thus, is reasonable to assume that estimates from the T–S curve may be often higher than or equal to the true richness in real‐world communities.

We have to remark that as for most of models, a theoretical definition of upper bounds for the T–S curve is impossible, and evidence from simulated and case study data cannot be considered as exhaustive proofs that the ensuing estimates are true upper bounds, as they cannot cover all possible real‐world scenarios. However, our approach is the first attempt, to our knowledge, allowing a context‐specific assessment of estimates when information on true species richness lacks and that, by exploiting the properties of the T–S curve and known higher taxon richness, may lead to identify, if not “true,” at least plausible upper limits in species richness over large areas.

## CONFLICT OF INTEREST

None declared.

## AUTHOR CONTRIBUTION

SB, AT, and KIU conceived the idea; SB, AT, AP, and DS carried out field work and analyzed samples; SB analyzed the data with the support of KIU and AT; SB led the writing of the manuscript with considerable improvements provided by AT and KIU; all authors critically reviewed the manuscript.
