# Supplementary material for: An approach based on the total‐species accumulation curve and higher taxon richness to estimate realistic upper limits in regional species richness
Source: Ecol Evol. 2017 Nov 29;8(1):405–15. doi: 10.1002/ece3.3570 (PMC5756853; doi:10.1002/ece3.3570)
Supplement: Supplementary file 1 [file ECE3-8-405-s001.doc]

**An approach based on the Total-Species accumulation curve and higher-taxon richness to estimate realistic upper limits in regional species richness**

Bevilacqua, S.^1,*^, Ugland, K.I.^2^, Plicanti, A.^1^, Scuderi, D.^3^, Terlizzi, A.^4,5^

*^1^Laboratory of Zoology and Marine Biology, Dep. of Biological and Environmental Sciences and Technologies, University of Salento, 73100 Lecce, Italy*

*^2^Dep. of Marine Biology, University of Oslo, Pb 1066 Blindern, Oslo, 0316, Norway*

*^3^I.I.S.S. "Ettore Majorana", 95048 Catania, Italy*

*^4^Dept. of Life Sciences, University of Trieste, 34127 Trieste, Italy*

*^5^Stazione Zoologica Anton Dohrn, 80121 Napoli, Italy*

*Corresponding author: stanislao.bevilacqua@unisalento.it

**Supplementary Information**

Additional material complementing the article “*An approach based on the Total-Species accumulation curve and higher-taxon richness to estimate realistic upper limits in regional species richness*” by Bevilacqua, S., Ugland, K.I., Scuderi, D., Plicanti, A. & Terlizzi, A. is provided in this section. Supplementary Information consists of:

**1 FIGURE**

**Figure S1.** Study area.

**5 TABLES**

**Table S1.** Summary of sampling design for subareas and habitats.

**Table S2.** Taxonomic list of mollusc species and families recorded.

**Table S3.** Summary of PERMDISP among samples across time.

**Table S4.** Inventory of mollusc families potentially present in the investigated area.

**Table S5.** Estimates of family and species richness from T–S curves and other estimators.

**6 APPENDICES**

**Appendix S1.** Performance of T–S curve accounting for spatial heterogeneity.

**Appendix S2.** Pooling of original small-scale samples.

**Appendix S3.** Sample size vs. expected fraction of common, intermediate, and rare species.

**Appendix S4.** Performance of T–S curve accounting for commonness and rarity of species.

**Appendix S5.** Simulated data (as separate .xls file).

**Appendix S6.** Real presence/absence data (as separate .xls file).

**Figure S1.** Study area with sampled subareas and habitat distribution (see also Table S1). The area was divided in three small bays by two rocky capes, and consisted of low sandy shores, except for the southern sector, which was characterized by rocky cliffs. Urban and industrial settlements were amassed in the northern sector, although a power plant was located at the southernmost boundary of the area. Three main sea bottom habitats were found, namely rocky reefs (RR), *Posidonia oceanica* seagrass beds (PB), and coralligenous outcrops (CO), which showed heterogeneous distributions. Seven subareas (S1-S7) were sampled during a 4-years monitoring program. Sampling scheme is reported below (see also Table S1).


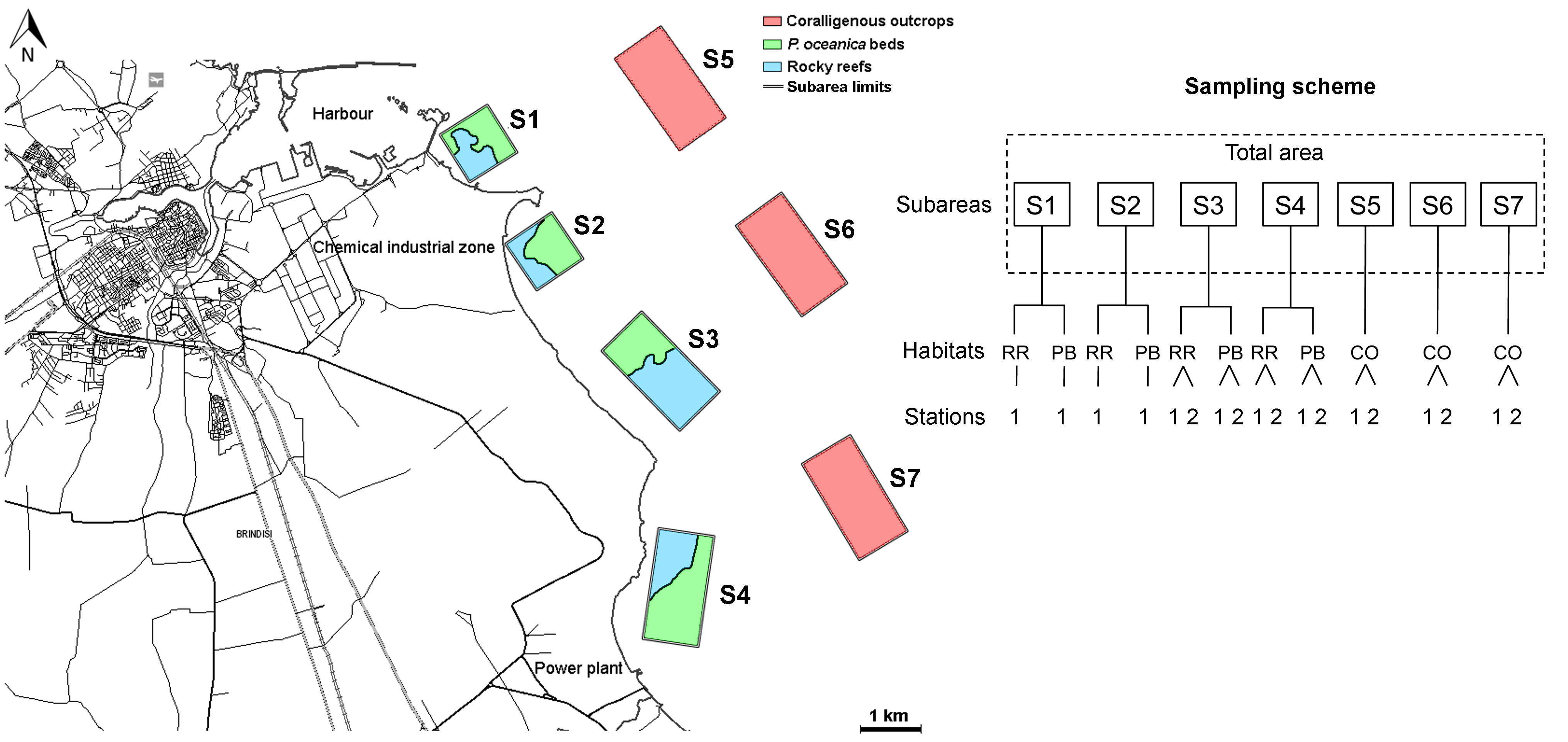


**Table S1. Sampling design for subareas and habitats. Subareas are named as in Fig. S1. RR = rocky reefs; PB = *Posidonia oceanica* beds; CO = coralligenous outcrops. Since subareas were not completely covered by the investigated habitats and included patches of sandy/detritic bottoms, the actual cumulative surface of sampled habitats within each subarea was provided. Estimates of family and species richness were extrapolated over the total surface of sampled habitats. Numbers in bold below the table are totals for each column. Sampling was carried out during a 4-years monitoring from 2010 until 2013. For subareas S1 and S2, a single station per year was sampled in each habitat (RR and PB). For subareas S3 and S4, two stations per year were sampled in each habitat (RR and PB). Finally, for subareas S5, S6 and S7, which were homogeneously characterized by a single habitat (CO), 2 stations per year were sampled. Each time of sampling, 3 replicates were sampled in each station.**

| **Subarea** | **Surface of subarea (m^2^)** | **Surface of sampled habitats within subarea (m^2^)** | **Sampled habitats** | **Subarea** × **Habitat spatial units** | **Sampling stations** | **Samples (replicates)** |
| --- | --- | --- | --- | --- | --- | --- |
| S1 | 1 × 10^6^ | 0.80 × 10^6^ | RR | S1 × RR | 4 | 12 |
|  |  |  | PB | S1 × PB | 4 | 12 |
| S2 | 1 × 10^6^ | 0.85 × 10^6^ | RR | S2 × RR | 4 | 12 |
|  |  |  | PB | S2 × PB | 4 | 12 |
| S3 | 2 × 10^6^ | 1.60 × 10^6^ | RR | S3 × RR | 8 | 24 |
|  |  |  | PB | S3 × PB | 8 | 24 |
| S4 | 2 × 10^6^ | 1.75 × 10^6^ | RR | S4 × RR | 8 | 24 |
|  |  |  | PB | S4 × PB | 8 | 24 |
| S5 | 2 × 10^6^ | 2 × 10^6^ | CO | S5 × CO | 8 | 24 |
|  |  |  |  |  |  |  |
| S6 | 2 × 10^6^ | 2 × 10^6^ | CO | S6 × CO | 8 | 24 |
|  |  |  |  |  |  |  |
| S7 | 2 × 10^6^ | 2 × 10^6^ | CO | S7 × CO | 8 | 24 |
|  |  |  |  |  |  |  |
| **7** | **12 × 10^6^** | **11 × 10^6^** | **3** | **11** | **72** | **216** |

**Table S2.** Taxonomic list of mollusc species and families recorded. Nomenclature followed Bedulli *et al.* (1995a), Bodon *et al.* (1995), Bedulli *et al.* (1995b), and Bedulli *et al.* (1995c), and was checked through the European Register of Marine Species database (http://www.marbef.org/data/erms.php).

| **Class** | **Family** | **Species** |
| --- | --- | --- |
| **POLYPLACOPHORA** | **ACANTHOCHITONIDAE** | *Acanthochitona crinita* (Pennant, 1977) |
|  |  | *Acanthochitona fascicularis* (Linnaeus, 1767) |
|  | **CHITONIDAE** | *Chiton* (*Rhyssoplax*) *olivaceus* (Spengler, 1797) |
|  | **ISCHNOCHITONIDAE** | *Ischnochiton* (*Ischnochiton*) *rissoi* (Payraudeau, 1826) |
|  | **LEPTOCHITONIDAE** | *Lepidopleurus cajetanus* (Poli, 1791) |
|  |  | *Leptochiton cimicoides* (Monterosato, 1879) |
|  | **CALLOCHITONIDAE** | *Callochiton septemvalvis* (Montagu, 1803) [ex *Callochiton euplaeae* (Costa O.G., 1829)] |
| **GASTROPODA** | **ANABATHRIDAE** | *Nodulus contortus* (Jeffreys, 1856) |
|  | **APLYSIIDAE** | *Petalifera petalifera* (Rang, 1828) |
|  | **BUCCINIDAE** | *Enginella leucozona* (Philippi, 1843) [ex *Engina leucozona*] |
|  |  | *Euthria cornea* (Linnaeus, 1758) [ex *Buccinulum corneum*] |
|  |  | *Pollia dorbignyi* (Payraudeau, 1826) |
|  | **BULLIDAE** | *Bulla striata* (Bruguière, 1792) |
|  | **CAECIDAE** | *Caecum clarkii* (Carpenter, 1858) |
|  |  | *Caecum glabrum* (Montagu, G., 1803) |
|  | **CALLIOSTOMATIDAE** | *Calliostoma laugieri* (Payraudeau, 1826) |
|  |  | *Calliostoma zyzyphinum* (Linnaeus, 1758) |
|  | **CALYPTRAEIDAE** | *Crepidula moulinsii* (Michaud, 1829) |
|  | **CERITHIIDAE** | *Bittium latreilli* (Payraudeau, 1826) |
|  |  | *Bittium reticulatum* (Da Costa, 1778) |
|  |  | *Bittium submamillatum* (De Rayneval & Ponzi, 1854) [ex *Cerithidium submamillatum*] |
|  |  | *Cerithium renovatum* (Monterosato, 1884) |
|  |  | *Cerithium repandum* (Monterosato, 1878) |
|  |  | *Cerithium vulgatum* (Bruguière, 1792) |
|  | **CERITHIOPSIDAE** | *Cerithiopsis buzzurroi* (Cecalupo & Robba, 2010) [ex *Nanopsis buzzurroi*] |
|  |  | *Cerithiopsis ladae* (Prkic & Buzzurro, 2007) |
|  |  | *Cerithiopsis minima* (Brusina, 1865) |
|  |  | *Cerithiopsis nana* (Jeffreys, 1867) [ex *Nanopsis nana*] |
|  |  | *Cerithiopsis tubercularis* (Montagu, 1803) |
|  |  | *Dizoniopsis coppolae* (Aradas, 1870) |
|  | **CHROMODORIDIDAE** | *Felimare gasconi* (Ortea, 1996) [ex *Hypselodoris gasconi*] |
|  | **CLATHURELLIDAE** | *Clathromangelia granum* (Philippi, 1844) |
|  | **COLLONIIDAE** | *Homalopoma sanguineum* (Linnaeus, 1758) |
|  | **COLUBRARIIDAE** | *Cumia reticulata* (Blainville, 1829) [ex *Colubraria reticulata*] |
|  | **COLUMBELLIDAE** | *Columbella rustica* (Linnaeus, 1758) |
|  | **CONIDAE** | *Conus ventricosus* (Gmelin, 1791) [ex *Conus mediterraneus* (Hwass in Bruguière, 1792)] |
|  | **CORNIROSTRIDAE** | *Tomura depressa* (Granata-Grillo, 1877) |
|  | **COSTELLARIIDAE** | *Vexillum* (*Pusia*) *ebenus* (Lamarck, 1811) |
|  |  | *Vexillum* (*Pusiolina*) *granum* (Forbes, 1844) [ex *Vexillum littorale*] |
|  |  | *Vexillum* (*Pusiolina*) *tricolor* (Gmelin, 1790) |
|  | **CYSTISCIDAE** | *Gibberula* *miliaria* (Linnaeus, 1758) |
|  |  | *Gibberula philippi* (Monterosato, 1878) |
|  | **DENDRODORIDIDAE** | *Dendrodoris limbata* (Cuvier, 1804) |
|  | **DORIDIDAE** | DORIDIDAE sp. |
|  | **EUBRANCHIDAE** | *Eubranchus* sp. (Forbes, 1838) |
|  | **EULIMIDAE** | *Eulima glabra* (Da Costa, 1778) |
|  |  | *Melanella boscii* (Payraudeau, 1827) |
|  |  | *Melanella sinuosa* (Scacchi, 1836) |
|  |  | *Parvioris ibizenca* (Nordsieck, 1968) |
|  |  | *Vitreolina philippi* (De Rayneval & Ponzi, 1854) |
|  | **FASCIOLARIIDAE** | *Fusinus syracusanus* (Linnaeus, 1758) |
|  | **FISSURELLIDAE** | *Diodora gibberula* (Lamarck, 1822) |
|  |  | *Emarginula huzardii* (Payraudeau, 1826) |
|  |  | *Emarginula octaviana* (Coen, 1939) |
|  | **HALIOTIDAE** | *Haliotis mykonosensis* (Owen, Hanavan & Hall, 2001) |
|  |  | *Haliotis tuberculata* (Linnaeus, 1758) [ex *Haliotis tuberculata tuberculata*] |
|  | **HAMINOEIDAE** | *Atys jeffreysi* (Weinkauff, 1866) |
|  |  | *Haminoea hydatis* (Linnaeus, 1758) |
|  |  | *Weinkauffia turgidula* (Forbes, 1844) |
|  | **HORAICLAVIDAE** | *Haedropleura septangularis* (Montagu, 1803) |
|  | **LIMIDAE** | *Lima lima* (Linneaus,1758) |
|  |  | *Limaria hians* (Gmelin, 1791) [ex *Lima hians*] |
|  | **MANGELIIDAE** | *Mangelia costulata* (Risso, 1826) |
|  |  | *Mangelia taeniata* (Deshayes, 1835) |
|  |  | *Mangelia unifasciata* (Deshayes, 1835) |
|  |  | *Mangelia vauquelini* (Payraudeau, 1826) |
|  | **MITRIDAE** | *Mitra cornea* (Lamarck, 1811) |
|  |  | *Mitra cornicula* (Linnaeus, 1758) |
|  | **MITROMORPHIDAE** | *Mitromorpha olivoidea* (Cantraine, 1835) [ex *Mitrolumna olivoidea*] |
|  | **MURICIDAE** | *Hexaplex trunculus* (Linnaeus, 1758) |
|  |  | *Muricopsis cristata* (Brocchi, 1814) [ex *Muricopsis inermis*] |
|  |  | *Ocinebrina aciculata* (Lamarck, 1822) |
|  |  | *Ocinebrina edwardsii* (Payraudeau, 1826) |
|  | **NASSARIIDAE** | *Nassarius cuvierii* (Payraudeau, 1826) |
|  |  | *Nassarius incrassatus* (Strom, 1768) |
|  |  | *Nassarius pygmaeus* (Lamarck, 1822) |
|  |  | *Nassarius unifasciatus* (Kiener, L.C., 1835) |
|  | **NATICIDAE** | *Notocochlis dillwynii* (Payraudeau, 1826) [ex *Natica dillwynii*] |
|  | **NERITIDAE** | *Smaragdia viridis* (Linnaeus, 1758) |
|  | **PHASIANELLIDAE** | *Tricolia pullus* (Linnaeus, 1758) [ex *Tricolia pullus pullus*] |
|  |  | *Tricolia speciosa* (Muhlfeld, 1824) |
|  |  | *Tricolia tenuis* (Michaud, 1829) |
|  | **PLAKOBRANCHIDAE** | *Elysia viridis* (Montagu, 1804) |
|  | **PLEUROBRANCHIDAE** | *Berthella plumula* (Montagu, 1803) |
|  | **PYRAMIDELLIDAE** | *Chrysallida excavata* (Philippi, 1836) [ex *Folinella excavata*] |
|  |  | *Eulimella acicula* (Philippi, 1836) |
|  |  | *Megastomia conoidea* (Brocchi, 1814) |
|  |  | *Odostomella doliolum* (Philippi, 1844) |
|  |  | *Odostomia eulimoides* Hanley, 1844 |
|  |  | *Odostomia fusulus* (Monterosato, 1878) |
|  |  | *Odostomia lukisii* (Jeffreys, 1859) |
|  |  | *Odostomia turrita* (Hanley, 1844) |
|  |  | *Odostomia wareni* (Schander, 1994) |
|  |  | *Parthenina emaciata* (Brusina, 1866) [ex *Chrysallida emaciata*] |
|  |  | *Parthenina indistincta* (Montagu, 1808) [ex *Chrysallida indistincta*] |
|  |  | *Parthenina interstincta* (Adams J., 1797) [ex *Chrysallida interstincta*] |
|  |  | *Parthenina terebellum* (Philippi, 1844) [ex *Chrysallida terebellum*] |
|  |  | *Pathenina incerta* (Milaschewitsch, 1916) [ex *Chrysallida incerta*] |
|  |  | *Turbonilla acutissima* (Monterosato, 1884) |
|  |  | *Turbonilla hamata* (Nordsieck, 1972) |
|  |  | *Turbonilla pusilla* (Philippi, 1844) |
|  |  | *Turbonilla striatula* (Linnaeus, 1758) |
|  | **RAPHITOMIDAE** | *Raphitoma horrida* (Monterosato, 1884) |
|  |  | *Raphitoma laviae* (Philippi, 1844) |
|  |  | *Raphitoma linearis* (Montagu, 1803) |
|  |  | *Raphitoma lineolata* (Bucquoy, Dautzenberg & Dollfus, 1883) |
|  |  | *Raphitoma philberti* (Michaud, 1829) |
|  |  | *Raphitoma pupoides* (Monterosato, 1884) |
|  |  | *Raphitoma purpurea* (Montagu, 1803) |
|  | **RETUSIDAE** | *Retusa laevisculpta* [ex *Cylichnina laevisculpt*a (Granata-Grillo, 1877)] |
|  |  | *Retusa truncatula* (Bruguière, 1792) |
|  | **RISSOELLIDAE** | *Rissoella inflata* (Locard, 1892) |
|  | **RISSOIDAE** | *Alvania cancellata* (Da Costa, 1778) |
|  |  | *Alvania carinata* (Da Costa, 1778) |
|  |  | *Alvania cimex* (Linnaeus, 1758) |
|  |  | *Alvania discors* (Allan, 1818) |
|  |  | *Alvania geryonia* (Nardo, 1847) |
|  |  | *Alvania hirta* (Monterosato, 1884) |
|  |  | *Alvania lineata* (Risso, 1826) |
|  |  | *Alvania mamillata* (Risso, 1826) |
|  |  | *Alvania pagodula* (Bucquoy, Dautzenberg & Dollfus,1884) |
|  |  | *Alvania scabra* (Philippi, 1844) |
|  |  | *Alvania settepassii* (Amati & Nofroni, 1985) |
|  |  | *Alvania subcrenulata* (Bucquoy, Dautzenberg & Dollfus, 1884) |
|  |  | *Crisilla semistriata* (Montagu, 1808) |
|  |  | *Manzonia crassa* (Kanmacher, 1798) |
|  |  | *Obtusella intersecta* (Wood, 1857) |
|  |  | *Pusillina inconspicua* (Alder, 1844) |
|  |  | *Pusillina marginata* (Michaud, 1832) |
|  |  | *Pusillina philippi* (Aradas & Maggiore, 1844) |
|  |  | *Pusillina radiata* (Philippi, 1836) |
|  |  | *Rissoa auriscalpium* (Linnaeus, 1758) |
|  |  | *Rissoa rodhensis* (Verduin, 1985) |
|  |  | *Rissoa similis* (Scacchi, 1836) |
|  |  | *Rissoa ventricosa* (Desmarest, 1814) |
|  |  | *Rissoa violacea* (Desmarest, 1814) |
|  |  | *Rissoina bruguieri* (Payraudeau, 1826) |
|  | **RUNCINIDAE** | *Runcina* sp. |
|  | **SCISSURELLIDAE** | *Scissurella costata* (D'Orbigny, 1824) |
|  | **SIPHONARIIDAE** | *Williamia gussoni* (Costa O. G., 1829) |
|  | **TRIPHORIDAE** | *Marshallora adversa* (Montagu, 1803) |
|  |  | *Metaxia metaxa* (Delle Chiaje, 1828) |
|  |  | *Monophorus thiriotae* (Bouchet, 1985) |
|  |  | *Similiphora similior* (Bouchet & Guillemot, 1978) |
|  | **TRITONIIDAE** | *Tritonia manicata* (Deshayes, 1853) |
|  | **TROCHIDAE** | *Clanculus corallinus* (Gmelin, 1791) |
|  |  | *Clanculus cruciatus* (Linnaeus, 1758) |
|  |  | *Clanculus jussieui* (Payraudeau, 1826) |
|  |  | *Gibbula adansoni* (Payraudeau, 1826) |
|  |  | *Gibbula adriatica* (Philippi, 1844) |
|  |  | *Gibbula ardens* (Von Salis, 1793) |
|  |  | *Gibbula turbinoides* (Deshayes, 1835) |
|  |  | *Gibbula umbilicaris* (Linnaeus, 1758) |
|  |  | *Jujubinus exasperatus* (Pennant, 1777) |
|  |  | *Jujubinus striatus* (Linnaeus, 1758) [ex *Jujubinus striatus striatus*] |
|  |  | *Jujubinus striatus fraterculus* (Monterosato, 1879) |
|  | **TURRITELLIDAE** | *Turritella turbona* (Monterosato, 1877) |
|  | **VERMETIDAE** | *Thylaeodus semisurrectus* (Bivona, 1832) [ex *Vermetus semisurrectus*] |
|  |  | *Vermetus triquetrus* (Bivona Ant., 1832) |
|  | **VOLVATELLIDAE** | *Ascobulla fragilis* (Jeffreys, 1856) |
| **BIVALVIA** | **ANOMIIDAE** | *Anomia ephippium* (Linnaeus, 1758) |
|  | **ARCIDAE** | *Arca noae* (Linnaeus, 1758) |
|  |  | *Barbatia barbata* (Linnaeus, 1758) |
|  | **CARDIIDAE** | *Papillicardium papillosum* (Poli, 1795) [ex *Plagiocardium papillosum*] |
|  |  | *Parvicardium exiguum* (Gmelin, 1791) |
|  |  | *Parvicardium pinnulatum* (Conrad, 1831) |
|  |  | *Parvicardium scabrum* (Philippi, 1844) |
|  |  | *Parvicardium scriptum* (Bucquoy, Dautzenberg & Dollfus,1892) |
|  | **CARDITIDAE** | *Cardita calyculata* (Linnaeus, 1758) |
|  |  | *Cardites antiquatus* (Linnaeus, 1758) [ex *Venericardia antiquata*] |
|  |  | *Glans trapezia* (Linnaeus, 1758) |
|  | **CHAMIDAE** | *Chama gryphoides* (Linnaeus, 1758) |
|  |  | *Pseudochama gryphina* (Lamarck, 1819) |
|  | **CLAVAGELLIDAE** | *Bryopa aperta* (= *Hiatella* sp.) (G.B. Sowerby I, 1823) [ex *Clavagella aperta*] |
|  | **CORBULIDAE** | *Corbula gibba* (Olivi, 1792) |
|  | **GALEOMMATIDAE** | *Galeomma turtoni* (Anonymous, 1825) |
|  | **GASTROCHAENIDAE** | *Rocellaria dubia* (Pennant, 1777) [ex *Gastrochaena dubia*] |
|  | **HIATELLIDAE** | *Hiatella arctica* (Linnaeus, 1767) |
|  | **KELLIIDAE** | *Bornia sebetia* (Costa O.G., 1829) |
|  |  | *Kellia suborbicularis* (Montagu, 1803) |
|  | **LASAEIDAE** | *Hemilepton nitidum* (Turton, 1822) |
|  | **LUCINIDAE** | *Ctena decussata* (Costa O.G., 1829) |
|  |  | *Loripes lucinalis* (Lamarck, 1818) |
|  |  | *Loripinus fragilis* (Philippi, 1836) [ex *Anodontia fragilis*] |
|  |  | *Lucinella divaricata* (Linnaeus, 1758) |
|  | **MACTRIDAE** | *Spisula subtruncata* (Da Costa, 1778) |
|  | **MONTACUTIDAE** | *Kurtiella bidentata* (Montagu, 1803) [ex *Mysella bidentata*] |
|  | **MYTILIDAE** | *Crenella arenaria* (Monterosato, 1875 ex H. Martin, ms) |
|  |  | *Crenella pellucida* (Jeffreys, 1850) |
|  |  | *Lithophaga lithophaga* (Linnaeus, 1758) |
|  |  | *Modiolula phaseolina* (Philippi, 1844) |
|  |  | *Modiolus barbatus* (Linnaeus, 1758) |
|  |  | *Musculus costulatus* (Risso, 1826) |
|  |  | *Musculus subpictus* (Cantraine, 1835) [ex *Modiolarca subpicta*] |
|  |  | *Mytilus galloprovincialis* (Lamarck, 1819) |
|  | **NOETIIDAE** | *Striarca lactea* (Linnaeus, 1758) |
|  | **NUCULIDAE** | *Austronucula perminima* (Monterosato, 1875) [ex *Nucula recondita* (Gofas & Salas, 1996)] |
|  |  | *Nucula hanleyi* (Winckworth, 1931) |
|  | **PECTINIDAE** | *Flexopecten hyalinus* (Poli, 1795) [ex *Chlamys hyalina*] |
|  |  | *Manupecten pesfelis* (Linnaeus, 1758) |
|  |  | *Mimachlamys varia* (Linnaeus, 1758) [ex *Chlamys varia*] |
|  |  | *Talochlamys multistriata* (Poli, 1795) [ex *Chlamys multistriata*] |
|  | **PSAMMOBIIDAE** | *Gari fervensis* (Gmelin, 1791) |
|  | **SEMELIDAE** | *Abra alba* (W. Wood, 1802) |
|  | **SPONDYLIDAE** | *Spondylus gaederopus* (juv) (Linnaeus, 1758) |
|  | **TELLINIDAE** | *Arcopagia balaustina* (Linnaeus, 1758) |
|  |  | *Moerella donacina* (Linnaeus, 1767) [ex *Tellina donacina*] |
|  |  | *Moerella pygmaea* (Lovén, 1846) [ex *Tellina pygmaea*] |
|  | **THRACIIDAE** | *Thracia distorta* (Montagu, 1803) |
|  |  | *Thracia phaseolina* (Poli, 1791) [ex *Thracia papyracea*] |
|  | **UNGULINIDAE** | *Diplodonta trigona* (Scacchi, 1835) [ex *Diplodonta apicalis* (Philippi, 1836)] |
|  | **VENERIDAE** | *Chamelea gallina* (Linnaeus, 1758) |
|  |  | *Gouldia minima* (Montagu, 1803) |
|  |  | *Irus irus* (Linneus, 1758) |
|  |  | *Lajonkairia lajonkairi*i (Payraudeau, 1826) [ex *Petricola lajonkairii*] |
|  |  | *Petricola lithophaga* (Retzius, 1786) |
|  |  | *Polititapes aureus* (Gmelin, 1791) [ex *Paphia aurea* - *Venerupsis aurea*] |
|  |  | *Venus verrucos*a (Linnaeus, 1758) |
| **CEPHALOPODA** | **SEPIOLIDAE** | *Sepiola* sp. |
| **SCAPHOPODA** | **DENTALIIDAE** | *Antalis vulgaris* (Da Costa, 1778) |

*References*

Bedulli, D., Castagnolo, L., Ghisotti F. & Spada, G. (1995a) Bivalvia, Scaphopoda. *Checklist delle specie della fauna italiana* (eds Minelli, A., Ruffo, S. & La Posta, S.), 17, pp. 1-21. Calderini, Bologna.

Bedulli, D., Cattaneo Vietti, R., Chemello, R., Ghisotti, F. & Giovine, F. (1995b) Gastropoda Opistobranchia, Divasibranchia, Gymnomorpha. *Checklist delle specie della fauna italiana* (eds Minelli, A., Ruffo, S. & La Posta, S.), 15, pp. 1-24. Calderini, Bologna.

Bedulli, D., Dell’Angelo, B & Salvini Plawen, L.V. (1995c) Caudofoveata, Solenogastres, Polyplacophora, Monoplacophora. *Checklist delle specie della fauna italiana* (eds Minelli, A., Ruffo, S. & La Posta, S.), 13, pp. 1-5. Calderini, Bologna.

Bodon, M., Favilli, L., Giannuzi Savelli, R., Giovine, F., Giusti, F., Manganelli, G., Melone, G., Oliverio, M., Sabelli, B. &. Spada, G. (1995) Gastropoda Prosobranchia, Heterobranchia Heterostropha. *Checklist delle specie della fauna italiana* (eds Minelli, A., Ruffo, S. & La Posta, S.), 14, pp. 1-60. Calderini, Bologna.

**Table S3.** Summary of tests for multivariate dispersion (PERMDISP) carried out to check for difference in β-diversity among groups of samples coming from different years. Tests were done for each Subarea × Habitat units, and were based on Jaccard's distance matrices among samples, with 999 permutations. Pair-wise tests among times of sampling are also reported when results of main tests were significant. T1 = 2010, T2 = 2011, T3 = 2012, T4 = 2013. Results showed that patterns of β-diversity were consistent through time in the vast majority of cases, thus excluding substantial effects of time in modifying spatial patterns of β-diversity in the whole sampled area.

| **Subarea** × **Habitat unit** | ***F*** | ***P*-perm** | **Pair-wise tests** |
| --- | --- | --- | --- |
| S1×RR | 5.425 | 0.09 | - |
| S1×PB | 1.738 | 0.58 | - |
| S2×RR | 9.168 | 0.03 | T1=T2=T3≠T4 |
| S2×PB | 0.518 | 0.80 | - |
| S3×RR | 2.436 | 0.20 | - |
| S3×PB | 1.597 | 0.32 | - |
| S4×RR | 0.153 | 0.96 | - |
| S4×PB | 1.218 | 0.35 | - |
| S5×CO | 0.455 | 0.78 | - |
| S6×CO | 2.112 | 0.22 | - |
| S7×CO | 3.922 | 0.03 | T1=T2≠T3=T4 |

**Table S4. Inventory of mollusc families that were potentially present in the investigated area. The inventory was mined integrating checklists of mollusc species at national and basin scale (Bedulli *et al*. 1995a,b,c; Bodon *et al.* 1995; Chiarelli, Tisselli Giunchi & Quadro, 1999; Storch 2003). A total of 242 families were selected, representing the maximum number of families in the whole area.**

| **Class** | **Family** | **Notes** |
| --- | --- | --- |
| Monoplacophora |  | The unique family, Neopilinidae, was excluded because typical of deep-sea habitats |
| Polyplacophora | Acanthochitonidae |  |
|  | Callistoplacidae |  |
|  | Callochitonidae |  |
|  | Chaetopleuridae |  |
|  | Chitonidae |  |
|  | Hanleyidae |  |
|  | Ischnochitonidae |  |
|  | Leptochitonidae |  |
| Caudofoveata | Chaetodermatidae | Limifossoridae and Prochaetodermatidae were excluded because typical of deep-sea habitats |
| Solenogastres | Lepidomeniidae | Amphimeniidae, Dondersiidae, Pruvotinidae and Rhopalomeniidae were excluded because typical of deep-sea habitats |
|  | Neomeniidae |  |
|  | Proneomeniidae |  |
|  | Simrothiellidae |  |
|  | Strophomeniidae |  |
| Gastropoda | Aclididae | Pelagic/planktonic (Atlantidae, Carinariidae, Cliopsidae, Cymbuliidae, Desmopteridae, Fionidae, Glaucidae, Janthinidae, Limacinidae, Notobranchaeidae, Peraclidae, Phylliroidae, Pneumodermatidae, Pterotracheidae, Scyllaeidae), brackish water (Potamididae), and deep-sea (Cocculinidae, Elachisinidae, Lepetellidae, Pseudocculinidae, Xylodisculidae) families were excluded. Families typical of supratidal and/or intertidal zones (Ellobiidae, Littorinidae, Onchidiidae, Truncatellidae) were also excluded. |
|  | Acmaeidae |  |
|  | Acteocinidae |  |
|  | Acteonidae |  |
|  | Addisoniidae |  |
|  | Aegiridae |  |
|  | Aeolidiidae |  |
|  | Aglajidae |  |
|  | Akeridae |  |
|  | Amathinidae |  |
|  | Anabathridae |  |
|  | Anatomidae |  |
|  | Aplysiidae |  |
|  | Aporrhaiidae |  |
|  | Architectonicidae |  |
|  | Arminidae |  |
|  | Asperspinidae |  |
|  | Assimineidae |  |
|  | Barleeiidae |  |
|  | Borsonidae |  |
|  | Bosellidae |  |
|  | Buccinidae |  |
|  | Bullidae |  |
|  | Bursidae |  |
|  | Cadlinidae |  |
|  | Caecidae |  |
|  | Caliphyllidae |  |
|  | Calliostomatidae |  |
|  | Calmidae |  |
|  | Calycidorididae |  |
|  | Calyptraeidae |  |
|  | Cancellariidae |  |
|  | Capulidae |  |
|  | Cassidae |  |
|  | Cavoliniidae |  |
|  | Cerithiidae |  |
|  | Cerithiopsidae |  |
|  | Chilodontidae |  |
|  | Chromodorididae |  |
|  | Cimidae |  |
|  | Cingulopsidae |  |
|  | Clathurellidae |  |
|  | Clavatulidae |  |
|  | Cliidae |  |
|  | Colloniidae |  |
|  | Colpodaspididae |  |
|  | Colubrariidae |  |
|  | Columbellidae |  |
|  | Conidae |  |
|  | Coralliophilinae |  |
|  | Cornirostridae |  |
|  | Costellariidae |  |
|  | Cylichnidae |  |
|  | Cypraeidae |  |
|  | Cystiscidae |  |
|  | Dendrodorididae |  |
|  | Dendronotidae |  |
|  | Dialidae |  |
|  | Diaphanidae |  |
|  | Discodorididae |  |
|  | Dorididae |  |
|  | Dotidae |  |
|  | Drilliidae |  |
|  | Embletoniidae |  |
|  | Epitoniidae |  |
|  | Eubranchidae |  |
|  | Eulimidae |  |
|  | Facelinidae |  |
|  | Fasciolaridae |  |
|  | Fissurellidae |  |
|  | Flabellinidae |  |
|  | Gastropteridae |  |
|  | Goniodorididae |  |
|  | Haliotidae |  |
|  | Haminoeidae |  |
|  | Hancockiidae |  |
|  | Hedylopsidae |  |
|  | Heroidae |  |
|  | Horaiclavidae |  |
|  | Hyalogyrinidae |  |
|  | Hydrobiidae |  |
|  | Iravadiidae |  |
|  | Lepetidae |  |
|  | Limapontiidae |  |
|  | Lomanotidae |  |
|  | Lottiidae |  |
|  | Madrellidae |  |
|  | Malleidae |  |
|  | Mangeliidae |  |
|  | Marginellidae |  |
|  | Mathildidae |  |
|  | Mitridae |  |
|  | Mitromorphidae |  |
|  | Mnestiidae |  |
|  | Murchisonellidae |  |
|  | Muricidae |  |
|  | Nassariidae |  |
|  | Naticidae |  |
|  | Neritidae |  |
|  | Notodiaphanidae |  |
|  | Omalogyridae |  |
|  | Onchidorididae |  |
|  | Orbistellidae |  |
|  | Ovulidae |  |
|  | Oxynoidae |  |
|  | Parhedylidae |  |
|  | Patellidae |  |
|  | Pendromidae |  |
|  | Phasianellidae |  |
|  | Philinidae |  |
|  | Philinoglossidae |  |
|  | Phyllidiidae |  |
|  | Piseinotecidae |  |
|  | Plakobranchidae |  |
|  | Planaxidae |  |
|  | Platyhedylidae |  |
|  | Pleurobranchaeidae |  |
|  | Pleurobranchidae |  |
|  | Polyceridae |  |
|  | Proctonotidae |  |
|  | Pseudovermidae |  |
|  | Pyramidellidae |  |
|  | Ranellidae |  |
|  | Raphitomidae |  |
|  | Retusidae |  |
|  | Rhizoridae |  |
|  | Rhodopidae |  |
|  | Ringiculidae |  |
|  | Rissoellidae |  |
|  | Rissoidae |  |
|  | Runcinidae |  |
|  | Scaliolidae |  |
|  | Scaphandridae |  |
|  | Scissurellidae |  |
|  | Siliquariidae |  |
|  | Siphonariidae |  |
|  | Skeneidae |  |
|  | Skeneopsidae |  |
|  | Strombidae |  |
|  | Tergipedidae |  |
|  | Tjaernoeiidae |  |
|  | Tofanellidae |  |
|  | Tonnidae |  |
|  | Tornidae |  |
|  | Trimusculidae |  |
|  | Triphoridae |  |
|  | Tritoniidae |  |
|  | Triviidae |  |
|  | Trochidae |  |
|  | Turbinidae |  |
|  | Turridae |  |
|  | Turritellidae |  |
|  | Tylodinidae |  |
|  | Umbraculidae |  |
|  | Vanikoridae |  |
|  | Velutinidae |  |
|  | Vermetidae |  |
|  | Volutidae |  |
|  | Volvatellidae |  |
|  | Xenophoridae |  |
| Bivalvia | Anomiidae | Deep-sea (Gryphaeidae, Neilonellidae, Trapeziidae, Xylophagidae) and brackish water (Dreissenidae) families were excluded |
|  | Arcidae |  |
|  | Astartidae |  |
|  | Basterotiidae |  |
|  | Cardiidae |  |
|  | Carditidae |  |
|  | Chamidae |  |
|  | Clavagellidae |  |
|  | Condylocardiidae |  |
|  | Corbulidae |  |
|  | Crassatellidae |  |
|  | Cuspidariidae |  |
|  | Donacidae |  |
|  | Galeommatidae |  |
|  | Gastrochaenidae |  |
|  | Glossidae |  |
|  | Glycymerididae |  |
|  | Hiatellidae |  |
|  | Kelliellidae |  |
|  | Kelliidae |  |
|  | Lasaeidae |  |
|  | Limidae |  |
|  | Limopsidae |  |
|  | Lucinidae |  |
|  | Lyonsiidae |  |
|  | Mactridae |  |
|  | Malletiidae |  |
|  | Mesodesmatidae |  |
|  | Montacutidae |  |
|  | Myidae |  |
|  | Mytilidae |  |
|  | Neoleptonidae |  |
|  | Noetidae |  |
|  | Nuculanidae |  |
|  | Nuculidae |  |
|  | Ostreidae |  |
|  | Pandoridae |  |
|  | Pectinidae |  |
|  | Periplomatidae |  |
|  | Pharidae |  |
|  | Phaseolidae |  |
|  | Pholadidae |  |
|  | Pholadomyidae |  |
|  | Pinnidae |  |
|  | Plicatulidae |  |
|  | Poromyidae |  |
|  | Psammobiidae |  |
|  | Pteriidae |  |
|  | Semelidae |  |
|  | Solecurtidae |  |
|  | Solemyidae |  |
|  | Solenidae |  |
|  | Spondylidae |  |
|  | Sportellidae |  |
|  | Tellinidae |  |
|  | Teredinidae |  |
|  | Thraciidae |  |
|  | Thyasiridae |  |
|  | Ungulinidae |  |
|  | Veneridae |  |
|  | Verticordiidae |  |
|  | Yoldiidae |  |
| Scaphopoda | Dentaliidae | Entalinidae and Pulsellidae were excluded because typical of deep-sea habitats |
|  | Fustiariidae |  |
|  | Gadilidae |  |
| Cephalopoda | Octopodidae | Most families (Ancistrocheiridae, Argonautidae, Bathyteuthidae, Brachioteuthidae, Chiroteuthidae, Chtenopterygidae, Cranchiidae, Cycloteuthidae, Enoploteuthidae, Histioteuthidae, Loliginidae, Octopoteuthidae, Ocythoidae, Ommastrephidae, Onychoteuthidae, Pyroteuthidae, Thysanoteuthidae, Tremoctopodidae) were excluded because mainly pelagic or typical of deep-sea habitats |
|  | Sepiidae |  |
|  | Sepiolidae |  |

*References*

Bedulli, D., Castagnolo, L., Ghisotti F. & Spada, G. (1995a) Bivalvia, Scaphopoda. *Checklist delle specie della fauna italiana* (eds Minelli, A., Ruffo, S. & La Posta, S.), 17, pp. 1-21. Calderini, Bologna.

Bedulli, D., Cattaneo Vietti, R., Chemello, R., Ghisotti, F. & Giovine, F. (1995b) Gastropoda Opistobranchia, Divasibranchia, Gymnomorpha. *Checklist delle specie della fauna italiana* (eds Minelli, A., Ruffo, S. & La Posta, S.), 15, pp. 1-24. Calderini, Bologna.

Bedulli, D., Dell’Angelo, B & Salvini Plawen, L.V. (1995c) Caudofoveata, Solenogastres, Polyplacophora, Monoplacophora. *Checklist delle specie della fauna italiana* (eds Minelli, A., Ruffo, S. & La Posta, S.), 13, pp. 1-5. Calderini, Bologna.

Bodon, M., Favilli, L., Giannuzi Savelli, R., Giovine, F., Giusti, F., Manganelli, G., Melone, G., Oliverio, M., Sabelli, B. &. Spada, G. (1995) Gastropoda Prosobranchia, Heterobranchia Heterostropha. *Checklist delle specie della fauna italiana* (eds Minelli, A., Ruffo, S. & La Posta, S.), 14, pp. 1-60. Calderini, Bologna.

Chiarelli, S., Tisselli Giunchi, M. & Quadri, P. (1999) *Nuovo catalogo delle conchiglie marine del Mediterraneo*. Società Italiana di Malacologia, 228 pp.

Storch, F. (2003) *Checklist of the species of the italian fauna*. Online version 2.0, Italian Ministry of Environment - Direction for Nature Protection. http://www.faunaitalia.it/checklist/index.html.

**Table S5. Estimates (±SD, standard deviation) of family and species richness based on T–S curves and other estimators. For T–S curves, reported steps correspond to those in Fig. 1 in the main text. A set of non-parametric (Chao1-2, Jackknife1), parametric asymptotic (negative exponential, Michaelis-Menten functions), and parametric non-asymptotic (Semi-log, Power functions) were applied. Maximum number of families is equal to 242; the observed number of families and species was 85 and 220 respectively.**

|  | **Families** | |  | **Species** | |
| --- | --- | --- | --- | --- | --- |
| **Estimator** | Estimate | SD |  | Estimate | SD |
| T-S curve Step 1 (original) | 302 | 18.1 |  | 945 | 24.8 |
| T-S curve Step 4 | 183 | 12.4 |  | 562 | 33.2 |
| Chao1 | 100 | 10.0 |  | 273 | 19.4 |
| Chao2 | 100 | 9.9 |  | 286 | 22.8 |
| Jackknife1 | 101 | 4.3 |  | 279 | 8.8 |
| Negative exponential | 79 | 69.1 |  | 208 | 137.6 |
| Michaelis-Menten | 89 | 34.1 |  | 250 | 69 |
| Semi-log | 275 | 8.8 |  | 805 | 83.8 |
| Power | 2032 | 33.5 |  | 17081 | 85.2 |

**Appendix S1.** Performance of T–S curve accounting for spatial heterogeneity.

The effect of heterogeneity in species distribution on species richness estimates from the T–S curve was explored by using simulated data. We simulated hypothetical areas having a total surface equal to 1,200 samples of size 1 (let say, for instance, 1 m^2^). Areas were projected to include 4 Spatial Units (SUs), each of them comprising 3 Subunits (SUBs), and a surface equal to 100 samples for each Subunit. A total of 250 species were distributed in different ways in hypothetical areas to simulate different scenarios of spatial heterogeneity. The occurrence of species in the 1,200 samples was also manipulated in order to obtain rare (frequency in samples <5%), intermediate (frequency >5% and <10%), and common (frequency >10%) species, following the classification provided by Gray & Ugland (1982).

Four main scenarios characterized by different patterns of heterogeneity in species composition within (among subunits) and among spatial units were created. Each scenario was reproduced for three different distributions of species among the previous categories of rarity, and namely, Most Rare (50% rare, 30% intermediate, and 20% common species), Even Distribution (1/3 rare, 1/3 intermediate, and 1/3 common species), Most Common (50% common, 30% intermediate, and 20% of rare species). A total of 12 simulated conditions, summarized in Table AS1-1, were thus obtained. Simulated data sets corresponding to the 12 conditions were provided in Appendix S5.

**Table AS1-1.** Main features characterizing simulated conditions. Similarity referred to species composition and was based on Jaccard’s index. Heterogeneity was calculated as multivariate dispersion among samples based on Jaccard’s distance to centroids and tested using PERMDISP (Anderson, 2006). MR = Most Rare, ED = Even Distribution, MC = Most Common.

| **Scenario of spatial heterogeneity** | Similarity among SUs | Similarity among SUBs within SUs | Variations in heterogeneity among SUs | Variations in heterogeneity among SUBs within SUs | Variations among SUs in overall heterogeneity of SUBs | **Pattern of rarity** | **Simulated condition** |
| --- | --- | --- | --- | --- | --- | --- | --- |
| **A (Very low)** | >70% | >70% | Not significant | Not significant | Not significant | **MR** | **#1** |
|  |  |  |  |  |  | **ED** | **#2** |
|  |  |  |  |  |  | **MC** | **#3** |
| **B (Low)** | 30%> >10% | <10% to >70% | Significant (p<0.05) | Not significant | Not significant | **MR** | **#4** |
|  |  |  |  |  |  | **ED** | **#5** |
|  |  |  |  |  |  | **MC** | **#6** |
| **C (High)** | <10% | >70% | Significant (p<0.05) | Significant (p<0.05) | Not significant | **MR** | **#7** |
|  |  |  |  |  |  | **ED** | **#8** |
|  |  |  |  |  |  | **MC** | **#9** |
| **D (Very high)** | <10% | <10% to >70% | Significant (p<0.05) | Significant (p<0.05) | Significant (p<0.05) | **MR** | **#10** |
|  |  |  |  |  |  | **ED** | **#11** |
|  |  |  |  |  |  | **MC** | **#12** |

For each simulated data set, 10 samples out of 100 were selected at random for each subunit, obtaining a subset of 120 random samples that, in practice, simulated a representative sampling of the hypothetical area (10% of the total surface). Simulated sampling was repeated 100 times, and a T–S curve based on the 4 SUs was built for each simulation in order to obtain 100 independent estimates of species richness. T–S curves was built on accumulation curves obtained using 100 random draws of the 120 samples for each combination of 1, 2, 3, 4 SUs (see Methods section for further details) and estimates referred to a total area *A* = 1,200.

The whole procedure was repeated in order to obtain also 100 independent estimates of species richness from T–S curves based on the 12 SUs×SUBs (i.e., taking into account both heterogeneity among spatial units and subunits). The average estimate (±95% CI, *n* = 100) of species richness from the two approaches was then calculated for each simulated condition. R script for simulated sampling is provided in Terlizzi *et al.* (2014).

Results highlighted that estimates obtained using SUs or SUs×SUBs to build the T–S curve largely overlapped only when spatial heterogeneity in species composition is very low (scenario A) whereas, as spatial heterogeneity among SUBs within SUs and among SUs increased (from scenario B to D), estimates from the two approaches differed increasingly (Table AS1-2, Fig. AS1-1). For each scenario, and irrespective of patterns of rarity, using SUs×SUBs to build the T–S curves led to improve the ensuing estimate of species richness (Table AS1-2).

**Table AS1-2.** Average species richness estimates from T–S curve based on spatial units (SUs) or spatial units × subunits (SUs×SUBs) under different simulated scenarios of spatial heterogeneity and patterns of rarity (see Table AS1-1). MR = Most Rare, ED = Even Distribution, MC = Most Common. Simulated conditions (#1 to #12) are also reported (see Table AS1-1). Numbers in brackets indicate % overestimation with respect to the total number of simulated species (250).

| **Scenario** |  | **A** | |  | **B** | |  | **C** | |  | **D** | |  |
| --- | --- | --- | --- | --- | --- | --- | --- | --- | --- | --- | --- | --- | --- |
| **T–S model** |  | SUs | SUs×SUBs |  | SUs | SUs×SUBs |  | SUs | SUs×SUBs |  | SUs | SUs×SUBs |  |
| **Rarity** | **MR** | 379(52%) | 386(54%) | **#1** | 457(83%) | 402(61%) | **#4** | 484(94%) | 413(65%) | **#7** | 461(84%) | 388(55%) | **#10** |
|  | **ED** | 362(45%) | 366(46%) | **#2** | 464(86%) | 409(64%) | **#5** | 469(88%) | 398(59%) | **#8** | 473(89%) | 402(61%) | **#11** |
|  | **MC** | 336(34%) | 333(33%) | **#3** | 469(88%) | 416(66%) | **#6** | 493(97%) | 420(68%) | **#9** | 483(93%) | 404(62%) | **#12** |


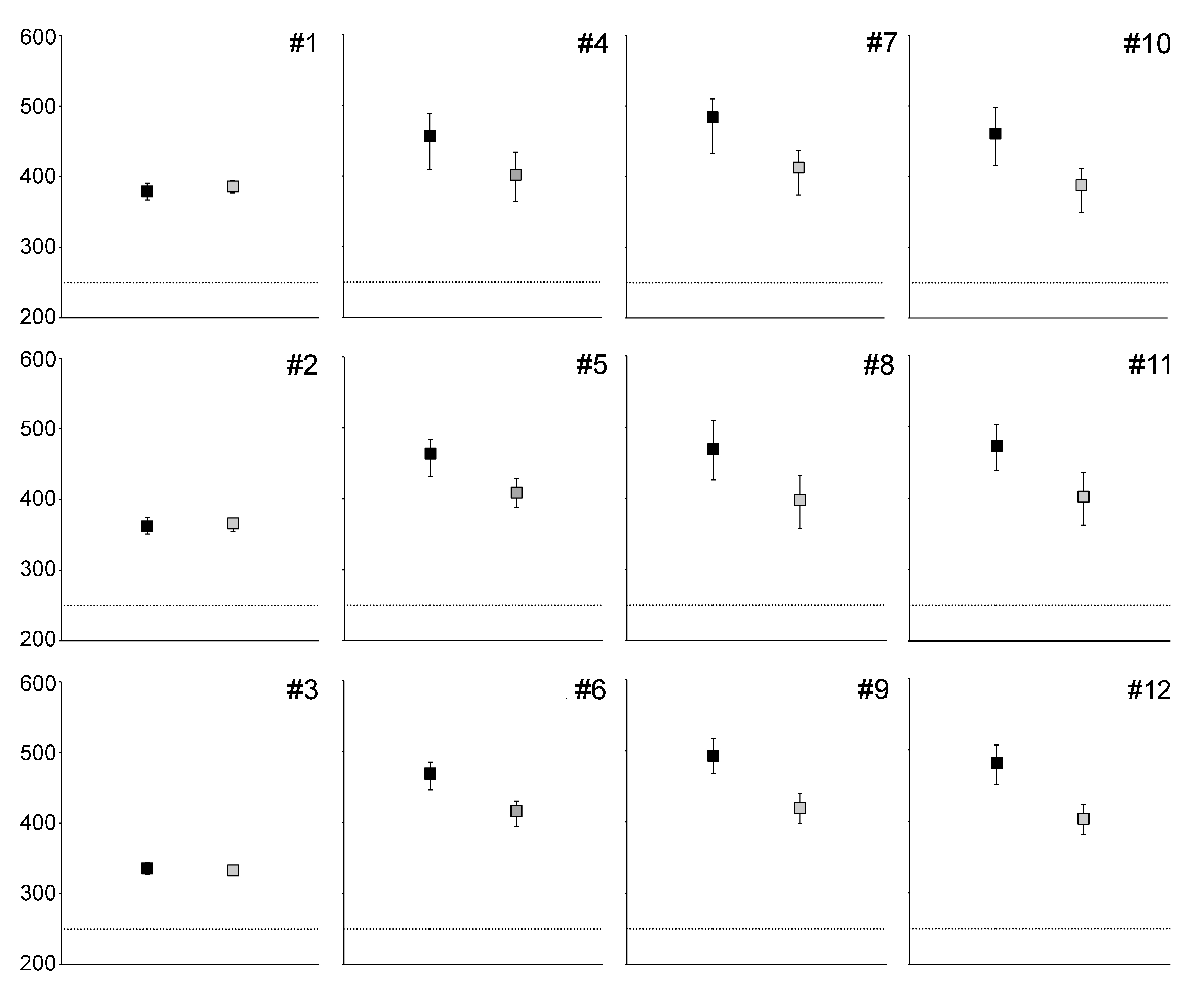


**Figure AS1-1.** Average (±95% CI, *n* = 100) estimated number of species (on *y*-axis) from T–S curves based on SUs (black squares) or SUs×SUBs (grey squares) under different simulated scenarios of spatial heterogeneity and patterns of rarity (see Table AS1-1). Dotted lines indicate the true species richness (250 species) in the hypothetical areas.

**Appendix S2.** Pooling of original smaller-scale samples.

Small-scale patchiness (i.e., the heterogeneity among replicate samples) in species composition could be partly the result of sample grain rather than of actual differences in species identities among assemblages that samples are supposed to represent. Therefore, we used the procedure proposed by Anderson & Santana-Garcon (2015) to identify the number of replicate samples that should be aggregated in order to quantify adequately species composition of local assemblages. The aim here is to obtain samples that provide sufficient information on species (or family) composition of local assemblages of interest (in our case mollusc assemblages). Replicate samples, if too small to capture adequately the assemblages of interest, may contain only a subset of species which is not representative of the larger-scale assemblages of interest. This may lead to (1) high rate of undefined or 100% (no species in common) pair-wise dissimilarities, and (2) skewed distributions of dissimilarity values. The procedure proposed by Anderson & Santana-Garcon (2015) uses simple graphs showing the behaviour of these two main characteristics of multivariate data as increasing number of replicate samples are pooled together, in order to guide the decision on sample aggregation.

The first analysis focused on the number of undefined and 100% dissimilarity values in the multivariate data. The original order of replicate samples was randomized (1,000 randomizations), plotting the mean (along with 0.025 and 0.975 quantiles) for the proportion of undefined dissimilarity values and the proportion of dissimilarity values equal to 100%, at increasing numbers of (randomized) replicate samples being used to form the 'new' sample. For the second analysis, increasing numbers of replicate samples were pooled in their original order and the distribution of pair-wise dissimilarity values was plotted. All analyses were based on Jaccard's dissimilarities and done separately for species and families. Results showed that, for both species and families, the aggregation of any *n* = 3 original smaller-scale samples (i.e., what we called 'replicate samples') excluded the presence of undefined and 100%-dissimilarity values (Fig. AS2-1). Moreover, the aggregation of the three replicates in each station substantially improved the distribution of pair-wise dissimilarities (Figg. AS2-2 and AS2-3). Therefore, pooling the three replicate samples in each station give a better representation of species composition of local assemblages.


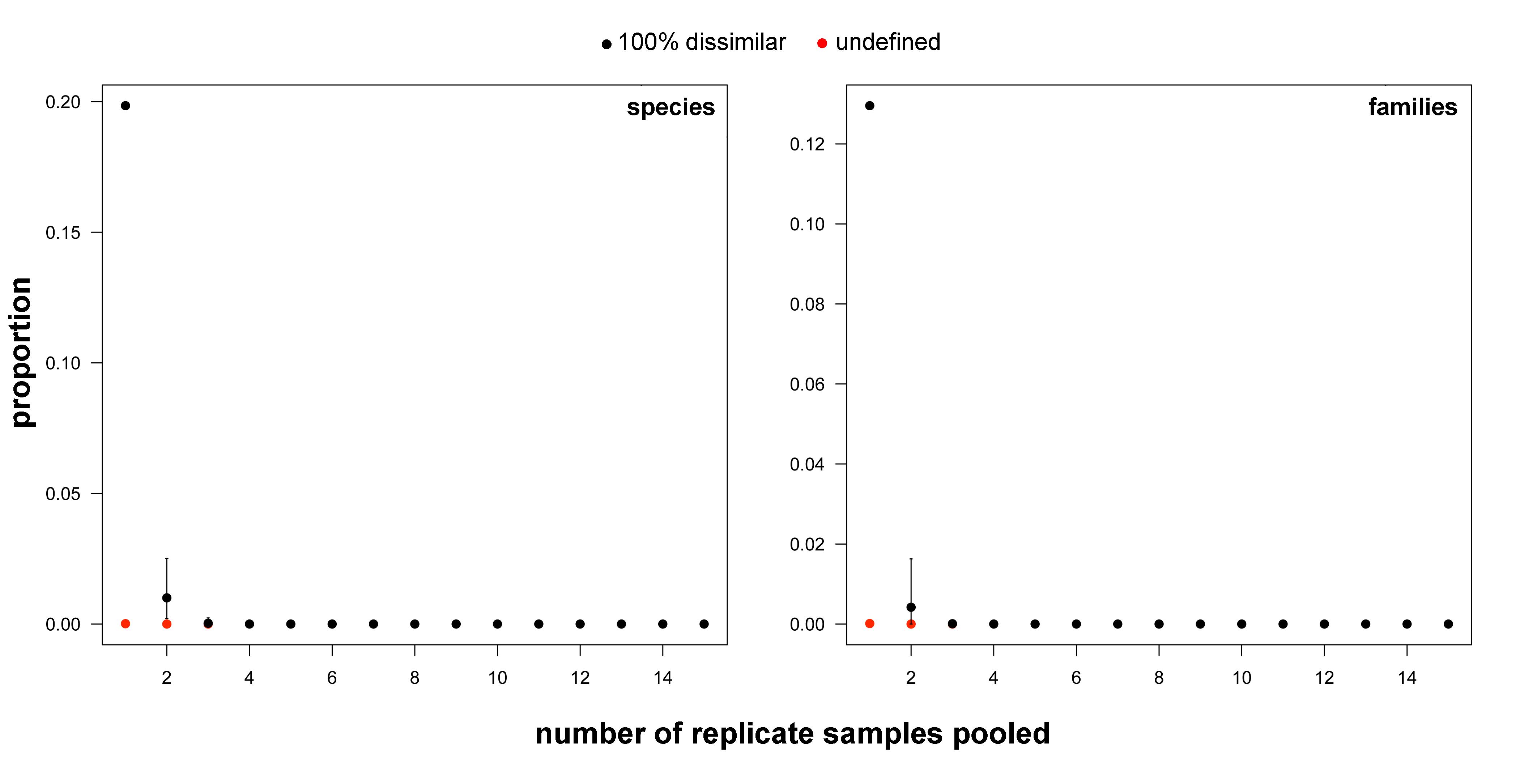


**Figure AS2-1.** Proportion of undefined and 100%-dissimilarity values in the multivariate data (real mollusc assemblages at species and family level) for increasing numbers of (randomized) original replicate samples being pooled to form the 'new' (aggregated) samples. Aggregations of >3 samples did not produce further improvements. Results for aggregations of >15 samples were not reported.


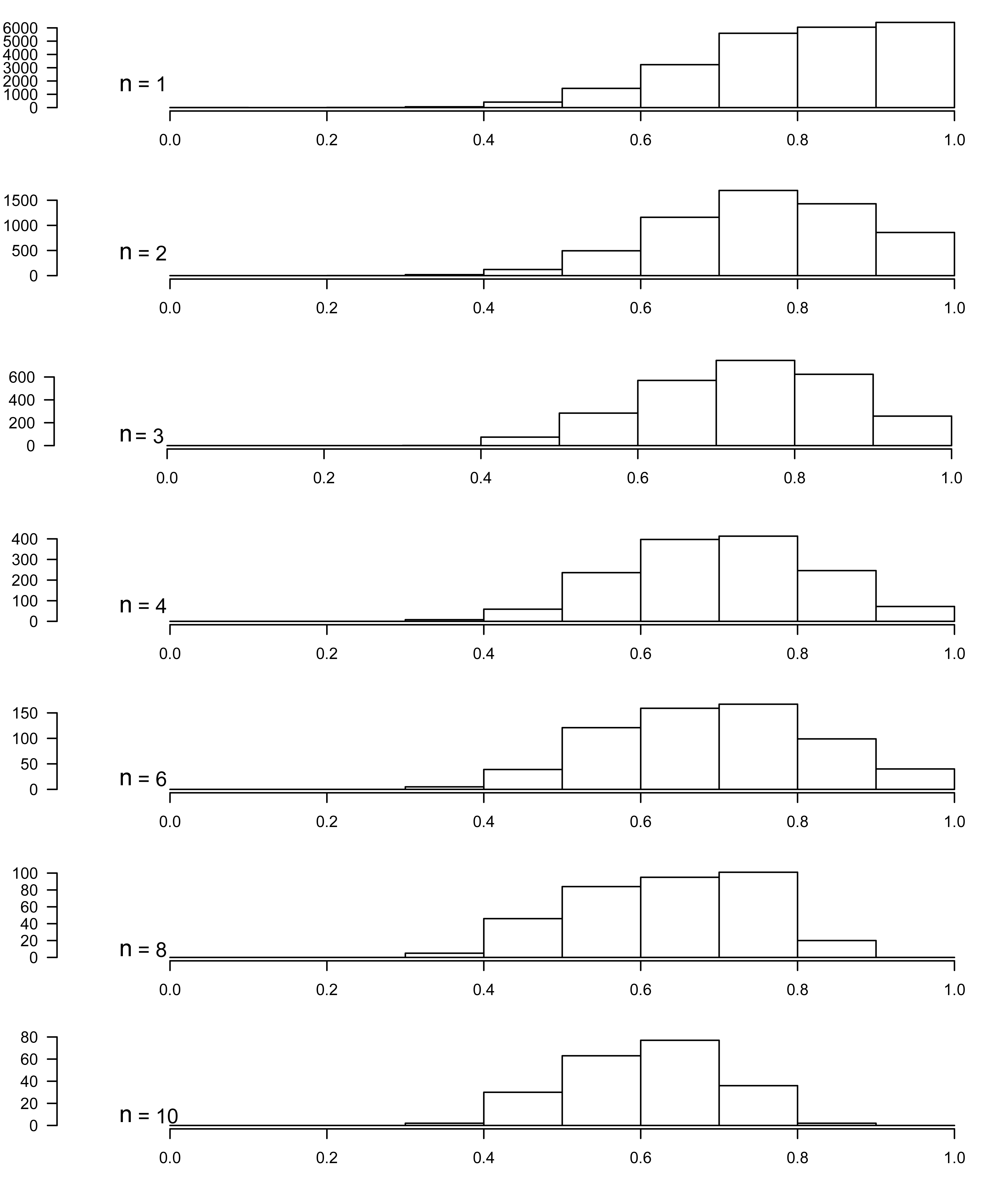


**Figure AS2-2.** Distribution of pair-wise dissimilarity values in the multivariate data (real mollusc assemblages at species level) for *n* = 1, 2, 3, 4, 6, 8, 10 original replicate samples being pooled (original order) to form the 'new' (aggregated) samples.


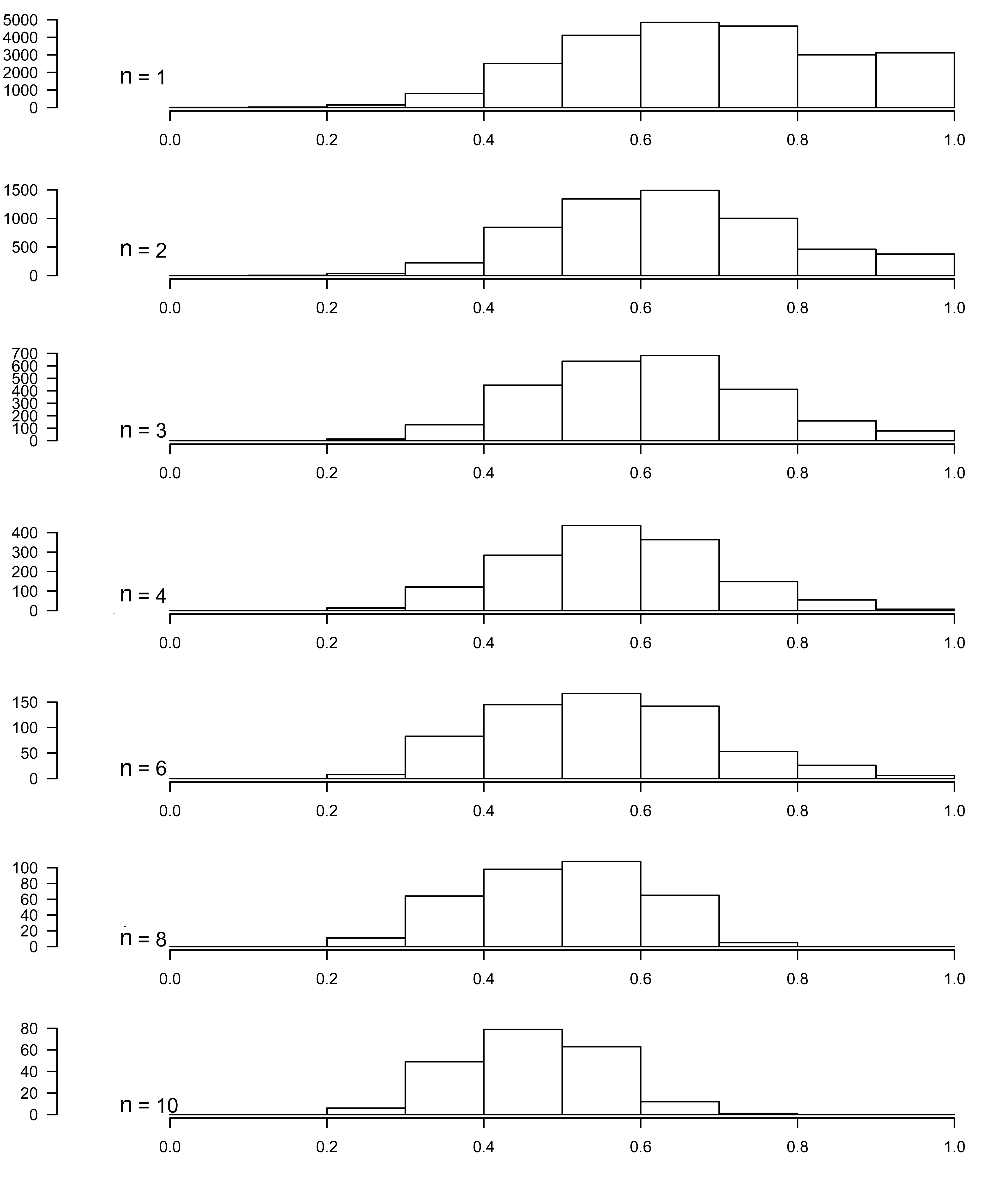


**Figure AS2-3.** Distribution of pair-wise dissimilarity values in the multivariate data (real mollusc assemblages at family level) for *n* = 1, 2, 3, 6, 8, 10 original replicate samples being pooled (original order) to form the 'new' (aggregated) samples.

**Appendix S3.** Sample size vs. expected fraction of common, intermediate, and rare species.

For a given set of *N* samples, common, intermediate and rare species are defined as those species found in >10%, between 5-10%, and <5% of samples respectively (see Methods section). Such definitions can be naturally applied also to genera, families, and more generally, to any taxonomic level.

Let’s focus on intermediate species (or taxa), for example, although the same logic can be extended also to common and rare species (or taxa). For simplicity, we considered discrete *p* values representative of the range of probability for intermediate species (i.e., 5% < *p* < 10%).

The probability of occurrence of an intermediate species *p* is one of the small alternatives *p* = 0.05, 0.06, 0.07, 0.08, 0.09, 0.10. The first random samples will therefore practically follow a binomial sequence with probability of success equal *p* in each trial. The probability of not seeing an intermediate species in *n* samples will therefore be equal to (1-*p*)*^n^*. The probability of observing an intermediate species in these first set of *n* samples is therefore equal to 1 - (1-*p*)*^n^*, for *n* =1, 2, ..., *N* in practically all ecological investigations. The higher the probability of occurrence, the higher the probability of observing the species in *n* samples. In the Table AS3-1 below is reported the exact number of samples in order to detect a given fraction of intermediate species (or taxa) for alternative values of *p*.

**Table AS3-1.** Number of samples required to detect a given fraction of intermediate species (or taxa) for alternative values of *p*.

| Expected fraction of intermediate species detected | Number of samples required at different values of *p* (i.e., the probability to detect intermediate species) | | | | | |
| --- | --- | --- | --- | --- | --- | --- |
|  | *p* = 0.05 | *p* = 0.06 | *p* = 0.07 | *p* = 0.08 | *p* = 0.09 | *p* = 0.10 |
| 75% | 27 | 23 | 19 | 17 | 15 | 13 |
| 80% | 32 | 26 | 22 | 19 | 17 | 15 |
| 85% | 37 | 31 | 26 | 23 | 20 | 18 |
| 90% | 45 | 37 | 32 | 28 | 25 | 22 |
| 95% | 59 | 49 | 41 | 36 | 32 | 29 |
| 99% | 90 | 75 | 64 | 56 | 49 | 44 |

A number of samples ranging between 44 and 90 will be therefore sufficient to detect a fraction of intermediate species (or taxa) ≥ 99%. The same sample size will be sufficient to detect the same (and even higher) fraction of common species, since they have a higher probability of occurrence (i.e., >10%, see above). Consequently, it is reasonable to assume that most, if not all, of the intermediate and common species (or taxa) in a given area of interest would be detected even after a relatively small portion of the area has been sampled, as occurs in most of ecological works. In contrast, due to their very low frequency, such an assumption is likely to be violated in the case of rare species, which probably require very high sampling efforts in order to be detected exhaustively.

In our specific case study, in which *N* = 72 samples (stations) were taken in the whole investigated area (see Methods section), the expected fraction of common, intermediate, and rare species (or taxa) for alternative values of *p* is reported in Table AS3-2.

**Table AS3-2.** Expected fraction of common, intermediate, and rare species (or taxa) for alternative values of *p*, calculated for *N* = 72 samples in our study case.

| **Common** | | | | | |
| --- | --- | --- | --- | --- | --- |
| *p* = 0.2 | *p* = 0.3 | *p* = 0.4 | *p* = 0.5 | *p =* 0.6 | *p* = 0.9 |
| 100% | 100% | 100% | 100% | 100% | 100% |
| **Intermediate** | | | | | |
| *p* = 0.05 | *p* = 0.06 | *p* = 0.07 | *p* = 0.08 | *p =* 0.09 | *p* = 0.1 |
| 98% | 99% | 99% | 100% | 100% | 100% |
| **Rare** | | | | | |
| *p* = 0.001 | *p =* 0.005 | *p* = 0.01 | *p* = 0.02 | *p* = 0.03 | *p* = 0.04 |
| 7% | 30% | 52% | 77% | 89% | 95% |

In this case, sample size appears large enough to detect all common and intermediate species (or families). It is expected, therefore, that accumulation curves for common and intermediate species and families are likely to reach the saturation, whereas this is unlikely to occur for rare ones.

Our choice to separate species into rare, intermediate and common (occurrence <5%, between 5% and 10%, >10% of samples respectively) is based on scientific literature (see Methods), and on the fact that these categories can be generally applied in the vast majority of studies. In fact, as showed in Table AS3-1, these three categories may apply with a good approximation (allowing detecting ≥99% of intermediate and 100% common species) to most studies with sufficient sampling effort (ranging between 44-90 samples). In our case, for instance, *N* = 72 samples allowed saturation (100%) of species with occurrence in samples ≥8% (Table AS3-2); since the fraction of detected species for *p* = 7%, 6%, 5% was 98% or higher, the saturation of species with occurrence ≥5% (i.e. intermediate [5%-10%] and common [>10%] species) can be reasonably assumed.

It is worth noting, however, that specific thresholds in species occurrence that could allow saturation (i.e. detection of 100% of species) can be derived for a given set of samples (Table AS3-3).

**Table AS3-3.** Thresholds of *p* for alternative number of samples.

| *p* | 0.1 | 0.085 | 0.073 | 0.065 | 0.058 | 0.051 | 0.035 | 0.027 | 0.021 | 0.018 | 0.016 | 0.014 | 0.012 | 0.01 |
| --- | --- | --- | --- | --- | --- | --- | --- | --- | --- | --- | --- | --- | --- | --- |
| No. of samples | 51 | 60 | 70 | 80 | 90 | 100 | 150 | 200 | 250 | 300 | 350 | 400 | 450 | 528 |

**Appendix S4.** Performance of T–S curve accounting for commonness and rarity of species.

The linear extrapolation of the T–S curve could cause overestimation since it implies that species richness increases continuously at increasing number of samples, whereas this could not be the case for accumulation of intermediate and common species, because they are likely to achieve saturation for sampling efforts commonly used in routine monitoring (see Appendix S3). Therefore, following eqn 4 (see Methods section), estimates of species richness may be improved by using an additive model in which the estimated number of rare species is summed to the number of observed common and intermediate species. We explored the performance of the additive model by using simulated data sets encompassing a wide range of conditions in terms of spatial heterogeneity and rarity of species (see Appendix S1), which are summarized below:

| **Simulated condition** | **Spatial heterogeneity** | **% of common, intermediate and rare species** |
| --- | --- | --- |
|  |  |  |
| #1 | Very low (Scenario A) | 20% common, 30% intermediate, 50% rare (Most Rare) |
| #2 | Very low (Scenario A) | 33% common, 33% intermediate, 33% rare (Even distribution) |
| #3 | Very low (Scenario A) | 50% common, 30% intermediate, 20% rare (Most Common) |
| #4 | Low (Scenario B) | 20% common, 30% intermediate, 50% rare (Most Rare) |
| #5 | Low (Scenario B) | 33% common, 33% intermediate, 33% rare (Even distribution) |
| #6 | Low (Scenario B) | 50% common, 30% intermediate, 20% rare (Most Common) |
| #7 | High (Scenario C) | 20% common, 30% intermediate, 50% rare (Most Rare) |
| #8 | High (Scenario C) | 33% common, 33% intermediate, 33% rare (Even distribution) |
| #9 | High (Scenario C) | 50% common, 30% intermediate, 20% rare (Most Common) |
| #10 | Very high (Scenario D) | 20% common, 30% intermediate, 50% rare (Most Rare) |
| #11 | Very high (Scenario D) | 33% common, 33% intermediate, 33% rare (Even distribution) |
| #12 | Very high (Scenario D) | 50% common, 30% intermediate, 20% rare (Most Common) |

We applied the same procedure detailed in Appendix S1 to obtain an average estimate (±95% CI, *n* = 100) of species richness from additive T–S models for each simulated condition. T–S curves for rare species were based on the 12 SUs×SUBs In all cases the number of samples (*n* = 120, see Appendix S1) allowed randomized accumulation curves for such common and intermediate species to achieve saturation. Estimates of species richness from additive models were then compared with those obtained from T–S curves based on the 12 SUs×SUBs, but considering all species. Results showed that, irrespective of different patterns of spatial heterogeneity and rarity, additive T–S models accounting for separated contributions of common, intermediate and rare species to total richness led to improved estimates of species richness (Table AS4-1), in most cases aligned with the true total richness in the hypothetical area (Fig. AS4-1).

**Table AS4-1.** Average species richness estimates under different scenarios of spatial heterogeneity and rarity of species (see Appendix S1) from T–S curves based on spatial units × subunits (SUs×SUBs) and all species, and additive T–S models to account for common, intermediate and rare species. MR = Most Rare, ED = Even Distribution, MC = Most Common. Simulated conditions (#1 to #12) are also reported (see above, see also Table AS1-1). Numbers in brackets indicate % overestimation with respect to the total number of simulated species (250).

| **Scenario** |  | **A** | |  | **B** | |  | **C** | |  | **D** | |  |
| --- | --- | --- | --- | --- | --- | --- | --- | --- | --- | --- | --- | --- | --- |
| **T–S model** |  | All species | Additive |  | All species | Additive |  | All species | Additive |  | All species | Additive |  |
| **Rarity** | **MR** | 386(54%) | 344(38%) | **#1** | 402(61%) | 313(25%) | **#4** | 413(65%) | 316(26%) | **#7** | 388(55%) | 287(15%) | **#10** |
|  | **ED** | 366(46%) | 307(23%) | **#2** | 409(64%) | 287(15%) | **#5** | 398(59%) | 266(6%) | **#8** | 402(61%) | 265(20%) | **#11** |
|  | **MC** | 333(33%) | 288(15%) | **#3** | 416(66%) | 270(8%) | **#6** | 420(68%) | 261(4%) | **#9** | 404(62%) | 253(1%) | **#12** |


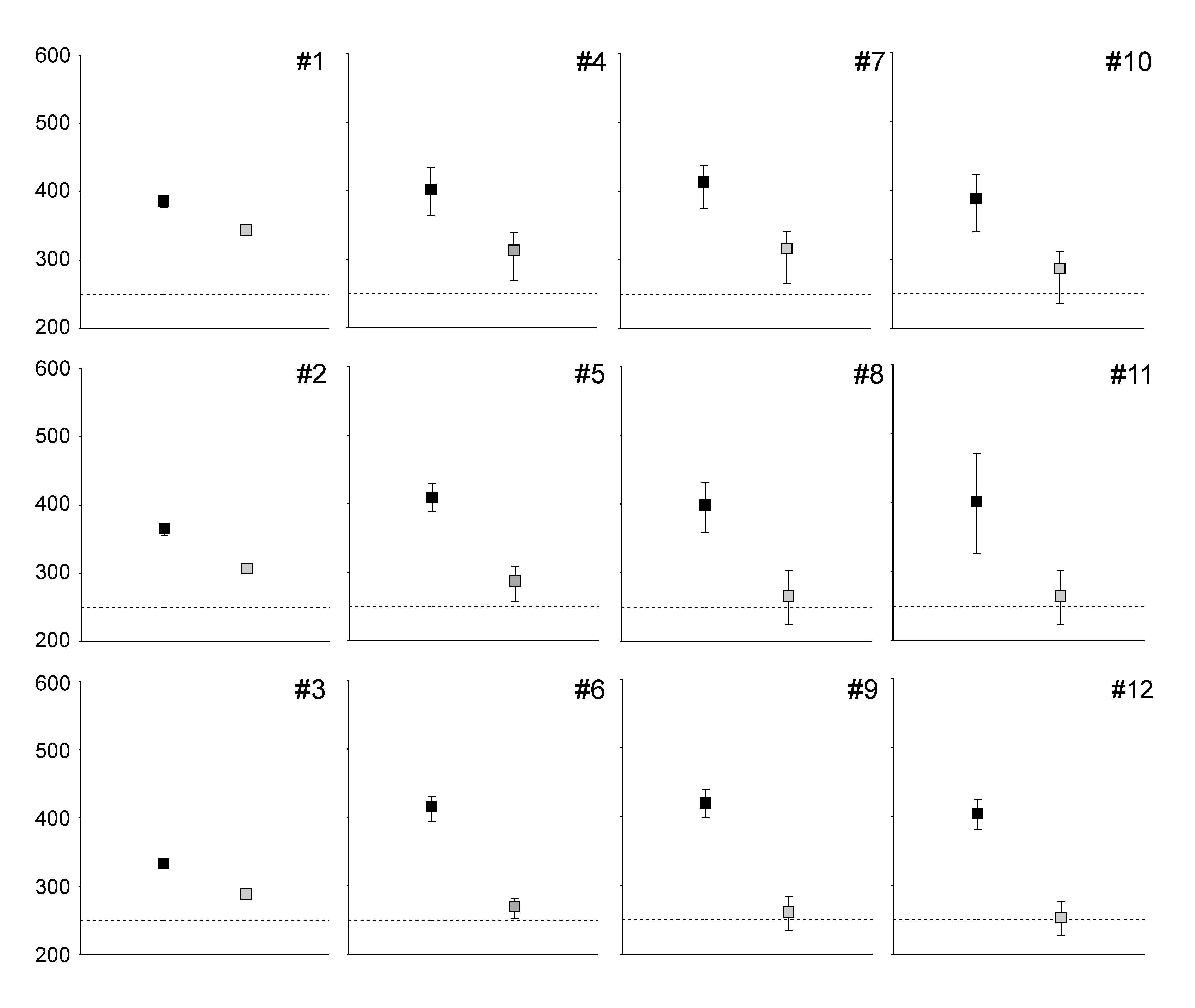


**Figure AS4-1.** Average (±95% CI, *n* = 100) estimated number of species (on *y*-axis) from T–S curves based on SUs×SUBs using all species (black squares) and the additive model to account separately for common, intermediate and rare species (grey squares), under different simulated scenarios of spatial heterogeneity and patterns of rarity (see Table AS1-1). Dotted lines indicate the true species richness (250 species) in the hypothetical areas.

**Appendix S5.** Simulated data (as separate .xls file).

**Appendix S6.** Real presence/absence data (as separate .xls file).
